# Multilocus phylogeny and cryptic diversity of white-toothed shrews (Mammalia, Eulipotyphla, *Crocidura*) in China

**DOI:** 10.1186/s12862-020-1588-8

**Published:** 2020-02-14

**Authors:** Shunde Chen, Jiao Qing, Zhu Liu, Yang Liu, Mingkun Tang, Robert W. Murphy, Yingting Pu, Xuming Wang, Keyi Tang, Keji Guo, Xuelong Jiang, Shaoying Liu

**Affiliations:** 1 College of Life Sciences, Sichuan Normal University, Chengdu, 610066 China; 2 Kunming Institute of Zoology, Chinese Academy of Sciences, Kunming, 650223 China; 3 College of Life Science and Technology, Mudanjiang Normal University, Mudanjiang, 157011 China; 4 Sichuan Academy of Forestry, Chengdu, 610081 China; 5 Centre for Biodiversity and Conservation Biology, Royal Ontario Museum, 100 Queen’s Park, Toronto, M5S 2C6 Canada; 6 Central South Forest Inventory and Planning Institute of State Forestry Administration, Changsha, 410014 China

**Keywords:** Mitochondrial DNA, Nuclear DNA, Rapid radiation, Soricidae, Species delimitation

## Abstract

**Background:**

*Crocidura*, the most speciose mammalian genus, occurs across much of Asia, Europe and Africa. The taxonomy of Chinese representatives has been studied primarily based on cursory morphological comparisons and their molecular phylogenetic analyses remain unexplored. In order to understand the phylogeny of this group in China, we estimated the first multilocus phylogeny and conducted species delimitation, including taxon sampling throughout their distribution range.

**Results:**

We obtained one mitochondrial gene (*cytb*) (~ 1, 134 bp) and three nuclear genes (*ApoB*, *BRCA1*, *RAG1*) (~ 2, 170 bp) for 132 samples from 57 localities. Molecular analyses identified at least 14 putative species that occur within two major well-supported groups in China. Polyphyletic *C. wuchihensis* appears to be composed of two putative species. Two subspecies, *C. rapax rapax* and *C. rapax kurodai* should be elevated to full species status. A phylogenetic tree based on mitochondrial gene from Asian *Crocidura* species showed that the *C. rapax rapax* is embedded within *C. attenuata*, making the latter a paraphyletic group. Three strongly supported undescribed species (*C.* sp.1, *C.* sp.2 and *C.* sp.3) are revealed from Zada County of Tibet (Western China), Hongjiang County of Hunan Province (Central China) and Dongyang County of Zhejiang Province (Eastern China), Motuo County of Tibet, respectively. The divergence time estimation suggested that China’s *Crocidura* species began to diversify during the late Pliocene (3.66 Ma) and the Early Pleistocene (2.29 Ma), followed by a series of diversifications through the Pleistocene.

**Conclusions:**

The cryptic diversity found in this study indicated that the number of species is strongly underestimated under the current taxonomy. We propose that the three undescribed species should be evaluated using extensive taxon sampling and comprehensive morphological and morphometric approaches. Climate change since the late Pliocene and the uplift of the Qinghai-Tibet Plateau may result in the diversification and speciation of China’s *Crocidura* species. In short, the underestimated diversity underlines the need for a taxonomic revision of Chinese *Crocidura* species.

## Background

*Crocidura* is the most speciose genus of all mammalian genera. Its 172 species occur broadly across much of Asia, Europe and Africa [1]. The highly conserved external morphologies of these species lead to their extreme taxonomic confusion [2, 3]. In China, species of *Crocidura* are among the most poorly understood mammals. The main challenge for taxonomy of Chinese *Crocidura* is the lack of available specimens. Some species and subspecies are only known from the holotypes. There are at least 19 recorded species and subspecies of *Crocidura* (Table 1). However, the taxonomic status and the number of species of Chinese *Crocidura* have changed over time (Table 1). For example, only 5 *Crocidura* species were recognized by Allen (1938) [4], while 8 species were listed by Honacki et al., 1982 [5]. Hutterer (1993) considered there were only 6 *Crocidura* species in China [6]. The current taxonomy recognizes 11 or 10 species [7, 8].

**Table 1:** Major classification systems of the genus *Crocidura* in China

Allen (1938) [4]	Honacki et al. (1982) [5]	Hutterer (1993) [6]	Jiang and Hoffmann (2001) [2]	Hutterer (2005) [1]	Hoffmann and Lunde (2008) [7]	Burgin and He (2018) [8]
*C. attenuata*	*C. attenuata*	*C. attenuata*	*C. attenuata*	*C. attenuata*	*C. attenuata*	*C. attenuata*
*–*	*–*	*–*	*–*	*C. tanakae*	*C. tanakae*	*C. tanakae*
*C. dracula*	*C. dracula*	*–*	*–*	*–*	*–*	*C. dracula*
*–*	*C. fuliginosa*	*C. fuliginosa*	*C. fuliginosa*	*C. fuliginosa*	*C. fuliginosa*	*–*
*–*	*C. horsfieldi*	*C. horsfieldii*	*C. horsfieldii wuchihensis*	*C. wuchihensis*	*C. wuchihensis*	*C. wuchihensis*
*–*	*–*	*–*	*C. horsfieldii indochinensis*	*C. indochinensis*	*C. indochinensis*	*C. indochinensis*
*–*	*C. lasiura*	*C. lasiura*	*C. lasiura*	*C. lasiura*	*C. lasiura*	*C. lasiura*
*C. rapax*	*C. russula*	*–*	*C. rapax*	*C. rapax*	*C. rapax*	*C. rapax*
*C. vorax*	*–*	*–*	*C. vorax*	*C. vorax*	*C. vorax*	*C. vorax*
*–*	*C. sibirica*	*C. sibirica*	*C. sibirica*	*C. sibirica*	*C. sibirica*	*–*
*–*	*C. suaveolens*	*C. suaveolens*	*–*	*–*	*–*	*C. suaveolens*
*C. ilensis*	*–*	*C. dsinezumi*	*C. gmelini*	*C. gmelini*	*C. gmelini*	*–*
	*–*		*C. shantungensis*	*C. shantungensis*	*C. shantungensis*	*C. shantungensis*

**Fig. 1:**
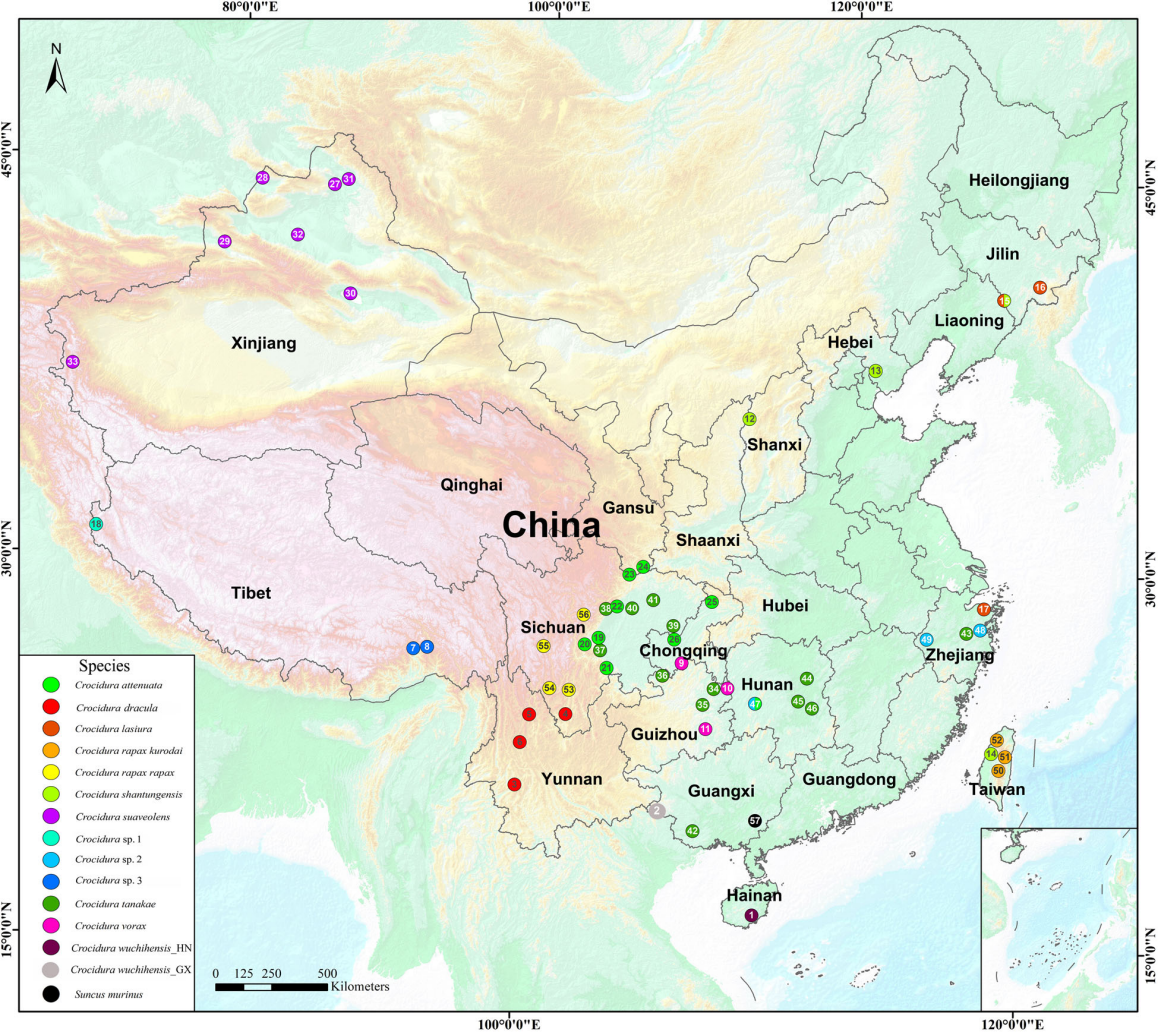
A map showing sample localities from China in this study. Different colors represent different species. The map was created by ArcGIS 10. 2 for Desktop. http://desktop.arcgis.com/zh-cn/arcmap

Chinese white-toothed shrews span Palearctic and Oriental faunal region (Fig. 1), but there have been only a few taxonomic studies of Chinese *Crocidura*. For example, one comprehensive morphological study enhanced the resolution of *Crocidura* from South China, and 6 species (*C. attenuata*, *C. fuliginosa*, *C. rapax*, *C. vorax*, *C. shantungensis* and *C. horsfieldii*) were recognized, and *C. vorax* and *C. rapax,* usually placed as synonyms of the European *C. russula,* are first recognized as 2 valid species [2]. Recently, a morphological and molecular study confirmed the presence of two white-toothed shrews (*C. lasiura* and *C. shantungensis*) in Northeast China [9]. The geographical distributions and morphological distinction of *C. tanakae* and *C. attenuata* were resolved based on a large number of specimens [10]. Compared with fewer studies in China, there are extensive studies of *Crocidura* in Southeast Asian countries and regions, such as in Vietnam [3, 11], Malaya and Indonesia [12–15], Philippines [16, 17] and Taiwan [17–19], Korea and Japan [20–23]. Although these previous studies did much to improve our understanding of the evolutionary relationships and taxonomy of *Crocidura* in Southeast Asian and regions, they rarely sampled species in China. The molecular phylogenetic relationships and genetic diversity of *Crocidura* within China remains largely unexplored.

Genetic investigations have often revealed systematic genetic relationships, as well as many cryptic species of small mammals, which is not surprising given their limited dispersal ability and morphological conservation [24–28]. Cryptic species of *Crocidura* may still exist within this widely distributed and diverse group of shrews [29–32], especially in the Qinghai-Tibetan Plateau and the Hengduan Mountains due to the extremely complex topography and variations in habitat [33].

In the last 20 years, we have collected more than 117 *Crocidura* specimens during our field surveys of small mammals through China. These specimens have expanded our knowledge concerning the distribution and habitats of *Crocidura*, but also allow us to re-examine the taxonomy of the genus in China. Herein, we evaluate extensive samples of *Crocidura* derived from China using a multilocus dataset and coalescent-based phylogenetic/species delimitation approaches. Our goals are (i) to determine if the diversity of Chinese *Crocidura* has been underestimated, (ii) to assess the phylogenetic relationships among these species, and (iii) to explore the evolutionary history of *Crocidura* in China.

## Results

### Sequence characteristics

We obtained 117 new *cytb* sequences [1, 134 bp], 55 *APOB* [522 bp], 55 *BRCA1* [793 bp] and 55 *RAG1* [855 bp] sequences (Table 2). No stop codon was observed in the coding regions of the protein-coding genes. The mitochondrial genes showed relatively more genetic polymorphism when compared with the three nuclear genes (Additional file 1: Table S1). Newly generated sequences were deposited in GenBank (MN690740-MN691036; Additional file 3: Table S3).

**Table 2:** Information of samples used from China in this study

Site	Detail Sample localities	Latitude	Longitude	Specimen Numbers	Species or subspecies	Source
1	Mt. Diaoluo, Hainan, China	18.722	109.868	3	*Crocidura wuchihensis*_HN	This study
2	Guangxi, China	23.116	105.966	3	*Crocidura wuchihensis*_GX	Genbank
3	Mt. Yongde, Yunnan, China	–	–	1	*Crocidura dracula*	Genbank
4	Miyi, Sichuan, China	26.997	101.874	3	*Crocidura dracula*	This study
5	Lijiang, Yunnan, China	26.927	100.229	3	*Crocidura dracula*	This study
6	Yangbi, Yunnan, China	25.793	99.871	3	*Crocidura dracula*	This study
7	Motuo, Xizang, China	29.244	95.015	2	*Crocidura* sp. 3	This study
8	Motuo, Xizang, China	29.326	95.326	3	*Crocidura* sp. 3	This study
9	Mt.Jinfo, Chongqing, China	29.049	107.194	1	*Crocidura vorax*	This study
10	Songtao, Guizhou, China	27.992	108.783	1	*Crocidura vorax*	This study
11	Leigongshan, Guizhou, China	26.385	108.203	1	*Crocidura vorax*	This study
12	Ningwu, Shanxi, China	38.633	111.037	1	*Crocidura shantungensis*	This study
13	Zunhua, Hebei, China	40.049	117.92	1	*Crocidura shantungensis*	This study
14	Taiwan, China	24.166	120.633	1	*Crocidura shantungensis*	Genbank
15	Xinbin, Liaoning, China	41.604	125.391	3	*Crocidura shantungensis*	This study
				3	*Crocidura lasiura*	This study
16	Fusong, Jilin, China	42.152	127.482	3	*Crocidura lasiura*	This study
17	Cixi, Zhejiang, China	–	–	1	*Crocidura lasiura*	Genbank
18	Zada, Xizang, China	31.783	78.872	1	*Crocidura* sp. 1	This study
19	Mingshan, Sichuan, China	30.076	103.178	3	*Crocidura attenuata*	This study
20	Yingjing, Sichuan, China	29.804	102.679	4	*Crocidura attenuata*	This study
21	Muchuan,Sichuan,China	28.876	103.714	1	*Crocidura attenuata*	This study
22	Pengzhou,Sichuan,China	31.314	103.852	1	*Crocidura attenuata*	This study
23	Tangjiahe, Sichuan,China	32.608	104.837	1	*Crocidura attenuata*	This study
24	Qingchuan,Sichuan,China	32.912	105.432	6	*Crocidura attenuata*	This study
25	Kaixian, Chongqing, China	31.455	108.712	1	*Crocidura attenuata*	This study
26	Huayingshan, Sichuan, China	30.388	106.847	1	*Crocidura attenuata*	This study
27	Fuhai, Xinjiang, China	47.075	87.427	2	*Crocidura suaveolens*	This study
28	Tacheng, Xinjiang, China	46.732	83.028	1	*Crocidura suaveolens*	This study
29	Yining, Xinjiang, China	43.911	81.731	1	*Crocidura suaveolens*	This study
30	Tulufan, Xinjiang, China	42.857	89.192	1	*Crocidura suaveolens*	This study
31	Aletai, Xinjiang, China	47.357	88.016	3	*Crocidura suaveolens*	This study
32	Mosuowan, Xinjiang, China	–	–	2	*Crocidura suaveolens*	Genbank
33	Tashenkuergan, Xinjiang, China	37.703	75.521	7	*Crocidura suaveolens*	This study
34	Mt. Fanjingshan, Guizhou, China	27.984	108.64	2	*Crocidura tanakae*	This study
35	Shiqian, Guizhou, China	27.364	108.11	1	*Crocidura tanakae*	This study
36	Hejiang, Sichuan, China	28.571	106.294	2	*Crocidura tanakae*	This study
37	Mt.Emei, Sichuan, China	29.571	103.399	2	*Crocidura tanakae*	This study
38	Dujiangyan, Sichuan, China	31.233	103.650	2	*Crocidura tanakae*	This study
39	Huayingshan, Sichuan,China	30.388	106.847	3	*Crocidura tanakae*	This study
40	Santai, Sichuan, China	31.261	104.898	1	*Crocidura tanakae*	This study
41	Langzhong, Sichuan,China	31.587	105.911	1	*Crocidura tanakae*	This study
42	Chongzuo, Guangxi,China	22.274	107.510	3	*Crocidura tanakae*	This study
43	Dongyang, Zhejiang, China	29.205	120.516	5	*Crocidura tanakae*	This study
44	Yuelushan, Hunan,China	28.189	112.945	3	*Crocidura tanakae*	This study
45	Hengshan, Hunan,China	27.280	112.691	2	*Crocidura tanakae*	This study
46	Hengdong, Hunan,China	26.970	113.067	1	*Crocidura tanakae*	This study
4	Hongjiang, Hunan,China	27.327	110.402	3	*Crocidura tanakae*	This study
				2	*Crocidura* sp. 2	This study
48	Dongyang, Zhejiang, China	29.205	120.516	7	*Crocidura* sp. 2	This study
49	Qiandaohu, Zhejiang, China	29.203	118.646	1	*Crocidura* sp. 2	This study
50	Nantou, Taiwan, China	23.668	120.988	3	*Crocidura rapax kurodai*	Genbank
51	Nantou, Taiwan, China	–	–	3	*Crocidura rapax kurodai*	Genbank
52	Taichung,Taiwan, China,	–	–	1	*Crocidura rapax kurodai*	Genbank
53	Jiulong, Sichuan, China	27.977	101.996	1	*Crocidura rapax rapax*	This study
54	Muli, Sichuan, China	28.019	101.102	2	*Crocidura rapax rapax*	This study
55	Yajiang, Sichuan, China	29.680	100.754	1	*Crocidura rapax rapax*	This study
56	Xiaojin, Sichuan, China	30.984	102.596	3	*Crocidura rapax rapax*	This study
57	Yulin, Guangxi, China	22.595	110.220	4	*Suncus murinus*	This study
Total				132		

### Phylogenetic analyses and molecular divergence times

**Fig. 2:**
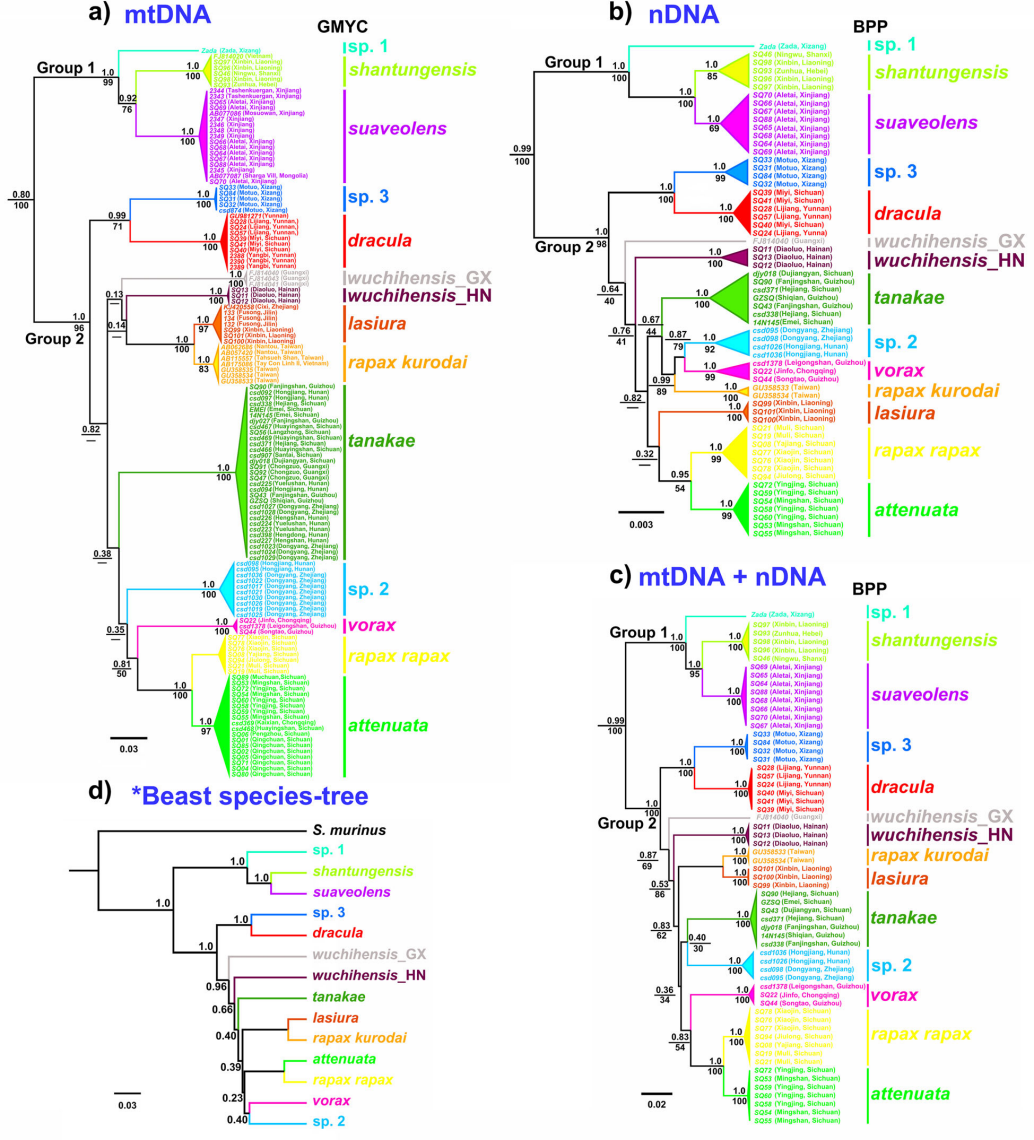
Results of Bayesian phylogenetic trees, species delimitation using splits and BPP, and species trees reconstructed using the *BEAST model. Bayesian phylogenetic trees derived from mtDNA dataset (**a**); nDNA dataset (**b**); mtDNA + nDNA dataset (**c**) and coalescent-based species tree using mtDNA + nDNA dataset (**d**). Numbers above branches refer to Bayesian posterior probabilities, and numbers below branches refer to the bootstrap values. Different colors represent different species in accordance with the Fig. 1

The phylogenies estimated by RAxML and BEAST were similar to each other for the first three molecular datasets (mtDNA, nDNA and mtDNA+ nDNA), and only the Bayesian Inference (BI) gene trees are shown (Fig. 2abc). In the BI and maximum likelihood (ML) trees, two well-supported major monophyletic groups were recovered from the first three molecular datasets (Fig. 2abc). One group was made up of *C. shantungensis*, *C. suaveolens* and *C.* sp.1 from Zada County of Tibet (Western China) and was strongly determined in all analyses (PP = 1.0, BS = 100). In the other group, phylogenetic relationships among species were not fully resolved (Fig. 2abc). *Crocidura dracula* was divided into two well-supported subclades. One subclade was from Motuo County of Tibet (*C.* sp. 3), and another subclade was from Yunnan and Sichuan (*C. dracula*) (Fig. 2abc). *Crocidura rapax* and *C. wuchihensis* appeared polyphyletic species*.* Subspecies *Crocidura r. kurodai* from Taiwan and *C. r. rapax* from Chongqing and Guizhou did not form a monophyletic clade, as did *C. wuchihensis* from Hainan (*C. wuchihensis*_HN) and *C. wuchihensis* from Guangxi and Vietnam (*C. wuchihensis*_GX) (Fig. 2abc). Several sister-species relationships were also revealed, such as *C. attenuata* and *C. r. rapax*, *Crocidura dracula* and *C.* sp. 3 (Fig. 2abc). Notably, an undescribed species (*C.* sp. 2) from Hongjiang County of Hunan Province (Central China) and Dongyang County of Zhejiang Province (Eastern China) was revealed. Its phylogenetic position has not been resolved in mtDNA and nDNA trees (Fig. 2ab).

**Fig. 3:**
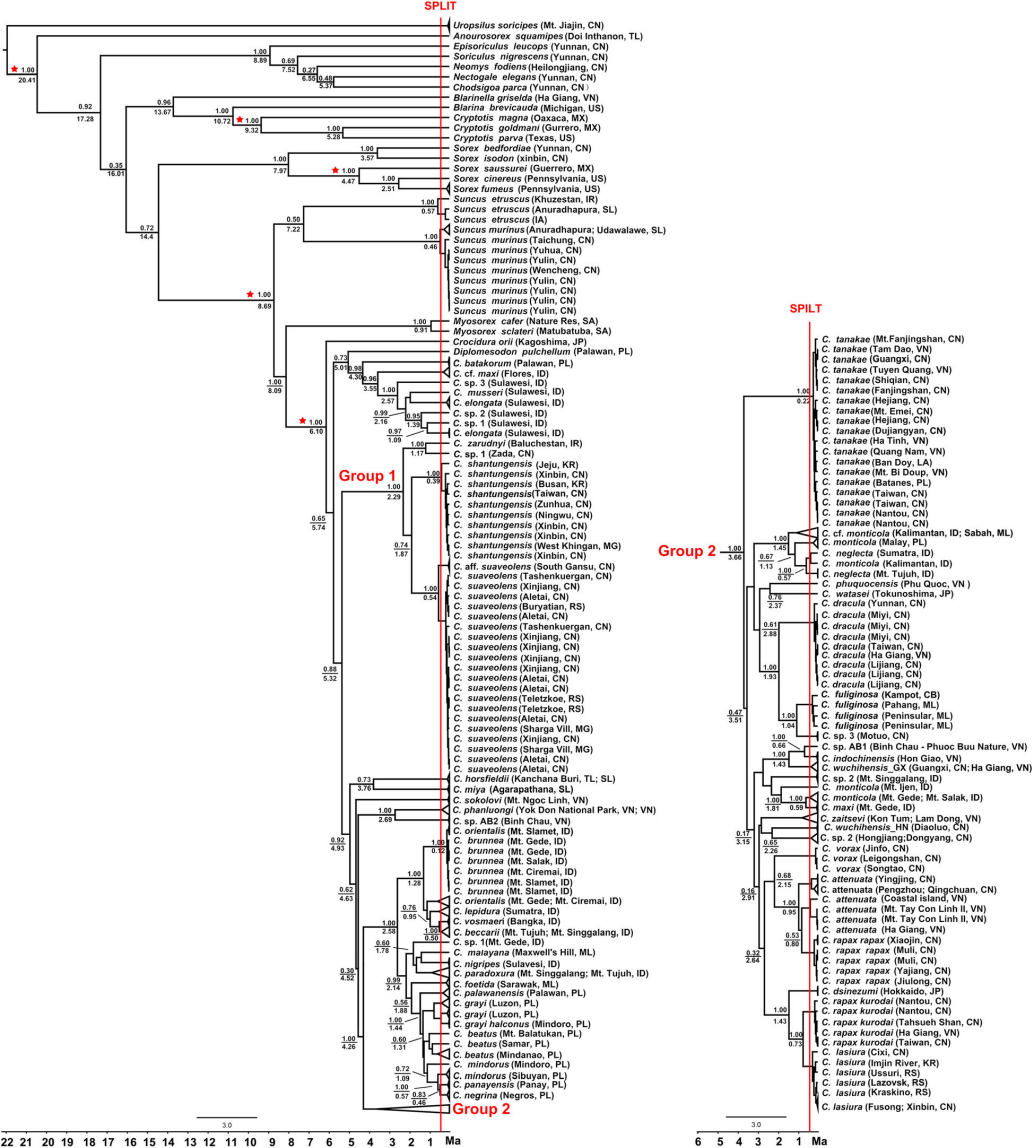
Chronogram from Bayesian analysis using a relaxed molecular clock and GMYC-based species delimitation based on dataset 4. The five red asterisks represent nodes whose age was calibrated with fossil taxa. Nodes of Bayesian posterior probabilities (above line) and estimated median times of divergence (below line) are shown. The red vertical lines indicate the maximum-likelihood transition point where the branching rates switch from interspecific to intraspecific events. Some triangles on tips are represented as the same species for simplification. Abbreviations of countries: America, US; Cambodia, CB; China, CN; India, IA; Indonesia, ID; Iran, IR; Japan, JP; Korea, KR; Laos, LA; Malaysia, ML; Mexico, MX; Mongolia, MG; Philippines, PL; Russia, RS; South Africa, SA; Sri Lanka, SL; Thailand, TL; Vietnam, VN

Phylogenetic relationships based on dataset 1(Fig. 2a) showed some differences compared to those obtained from datasets 4 (Fig. 3). For example, China’s *Crocidura* species is a not monophyletic group, including some *Crocidura* undescribed species/species from other countries, such as *C. zarudnyi* from Iran, *C. indochinensis*, *C. fuliginosa* and *C. zaitsevi* from Vietnam, *C. dsinezumi* from Japan, and *C. monticola*, *C. neglecta*, *C. maxi* from Indonesia and Malaysia (Fig. 3). The *C. fuliginosa* complex was divided into three well-supported subclades, including *C.* sp. 3, *C. dracula* and *C. fuliginosa*. When the *C. attenuata* samples from Vietnam were added, the phylogenetic tree showed that *C. r. rapax* was embedded within *C. attenuata*, making the latter a paraphyletic group (Fig. 3).

Divergence time estimates inferred that the most recent common ancestor (MRCA) of Asian *Crocidura* originated in the late Miocene period (6.10 Ma, highest posterior density (HPD) interval containing 95% of the sampled values: 4.72–7.69) (Fig. 3). China’s *Crocidura* species began to diversify during the late Pliocene (3.66 Ma) and the Early Pleistocene (2.29 Ma), followed by a series of diversifications through the Pleistocene (Fig. 3). The *C.* sp. 1 diverged from *C. zarudnyi* approximately 1.17 Ma (95% CI: 1.93–0.58). The *C. suaveolens* diverged from *C.* aff*. Suaveolens* approximately 0.54 Ma (95% CI: 0.89–0.27). The *C.* sp. 2 diverged from *C. wuchihensis*_HN approximately 2.26 Ma (95% CI: 3.19–1.44). The *C.* sp. 3 diverged from *C. fuliginosa* approximately 1.04 Ma (95% CI: 1.63–0.60) and *C. dracula* approximately 1.93 Ma (95% CI, 2.80–1.26).

### Species delimitation and species-trees

The GMYC and BPP analyses gave identical results. GMYC species delimitation analysis recognized 14 (confidence interval [CI] = 14–16) putative species based on dataset 1 (mtDNA) (Fig. 2a). When using either dataset 2 (nDNA) or dataset 3 (mtDNA + nDNA), BPP consistently supported the validity of 14 hypothetical species with high posterior probabilities (PP ≥ 0.94; Additional file 2: Table S2). When using the time-calibrated gene tree as the input tree derived from dataset 4, SPLITS suggested that clades with divergence times earlier than 0.418 Ma may have represented genetically distinct species (Fig. 3). In addition to supporting the 14 hypothetical species mentioned above, SPLITS also supported *C. indochinesis*, *C*. aff. *Suaveolens* and another *C. attenuata* population from Vietnam as a separate species (Fig. 3).

The *BEAST species-tree strongly divided species of *Crocidura* into two same groups as the concatenated and CYTB trees (Fig. 2ab), although the relationships within each group were not fully resolved. *C. attenuata* and *C. r. rapax*, *C. dracula* and *C.* sp. 3 were strongly supported as sister-taxa (PP = 1.0), as were *C. r. kurodai* and *C. lasiura* (PP = 1.0) (Fig. 2d).

## Discussion

### Mitochondrial and nuclear gene-trees discordance

Our gene-trees inferred some conflicting or poorly supported phylogenetic relationships within Chinese *Crocidura*, and the molecular genetic interrelationships among several species are not consistent in mtDNA and nDNA trees (Fig. 2ab). This discordance could be attributed to explosive speciation [34], incomplete lineage sorting [35] and introgression [36–38]. Explosive speciation (i.e., rapid radiation) offers an explanation for the discrepancy owing to the very short branches that have poor support (Fig. 2). Our time-calibrated Bayesian analysis indicates rapid cladogenesis from the late Pliocene to the Early Pleistocene (Fig. 3), when most diversification occurred. Such rapid speciation can preclude high resolution of the phylogenetic trees because informative DNA substitutions will not have time to accumulate (e.g., [39, 40]). A second explanation is that relatively recent radiation may not have provided enough time for a complete lineage sorting, further blurring the phylogenetic relationships of these species [41]. For example, there is a large genetic distance between *C.* sp. 2 and *C. vorax* and do not cluster together in mtDNA trees (Table 3), while relatively strong support for the clade *C.* sp. 2 + *C. vorax* in nDNA tree. However, distinguishing the two hypotheses will require additional nuclear loci [35, 42]. Another explanation for the discrepancy is an old introgression event, as reported in other shrews [37, 38]. For example, there is strong support for the clade *C. lasuria* + *C. kurodai* in mtDNA analysis and a small genetic distance between these two species (Table 3). However, these two species are very different and do not cluster together in nDNA trees.

**Table 3:** The *p*-distance among undescribed species/species of *Crocidura* based on the *cytb* gene

	*C. suaveolens*	*C. dracula*	*C. rapax kurodai*	*C. attenuata*	*C. shantungensis*	*C. wuchihensis_*GX	*C. lasiura*	*C. rapax rapax*	*C.* sp. 1	*C.* sp. 2	*C.* sp. 3	*C. tanakae*	*C. vorax*
*C. dracula*	0.138												
*C. rapax kurodai*	0.140	0.119											
*C. attenuata*	0.138	0.121	0.108										
*C. shantungensis*	0.084	0.140	0.134	0.141									
*C. wuchihensis_*GX	0.144	0.127	0.115	0.116	0.145								
*C. lasiura*	0.143	0.124	0.039	0.105	0.142	0.118							
*C. rapax rapax*	0.142	0.126	0.108	0.049	0.149	0.108	0.111						
*C.* sp. 1	0.088	0.138	0.136	0.139	0.093	0.132	0.137	0.142					
*C.* sp. 2	0.147	0.121	0.102	0.110	0.139	0.119	0.102	0.110	0.143				
*C.* sp. 3	0.139	0.097	0.110	0.124	0.131	0.125	0.114	0.121	0.127	0.118			
*C. tanakae*	0.134	0.116	0.105	0.120	0.151	0.121	0.107	0.115	0.142	0.114	0.117		
*C. vorax*	0.147	0.119	0.106	0.105	0.148	0.126	0.112	0.101	0.145	0.112	0.112	0.122	
*C. wuchihensis_*HN	0.141	0.115	0.112	0.115	0.143	0.121	0.118	0.110	0.136	0.104	0.107	0.110	0.115

### Phylogeny and rapid radiation among China’s *Crocidura* species

Our fine-scale sampling of *Crocidura* from China reveals two well-supported major groups of *Crocidura* (Fig. 2). Some species in the group 1 seemingly represents north-western *Crocidura* species, while Some species in the group 2 represents south-eastern *Crocidura* species in China (Fig. 2). However, the phylogenetic relationships among many species in the group 2 remain unresolved (Fig. 2). To resolve phylogenetic relationships in recent, rapid radiation group, such as *Crocidura*, is a challenge task. Previous phylogenetic studies of Philippine *Crocidura* using multilocus data also yielded many poorly supported branches [14, 43]. Fortunately, phylogenomic analyses often have the potential to resolve difficult phylogenetic relationships [44, 45]. However, using hundreds of ultraconserved elements (UCEs) and whole mitogenomes, Giarla and Esselstyn [46] could not fully resolve the phylogenetic relationships among the Philippine *Crocidura* based on coalescent-based approaches. Restriction site associated DNA sequencing (RADseq) is a commonly used approach for phylogeny estimation, and has been proved to be effective in solving some difficult phylogenetic relationships [45, 47, 48]. However, this RADseq could also lead to bias phylogenetic relationships when locus filtering techniques can not accurately identify homologous loci [46, 49]. Thus, to completely resolve phylogenetic relationships using rapid high-throughput sequencing technology for this recent, rapid radiation *Crocidura* group may be still go a long way.

According to our divergence time estimation, China *Crocidura* species have rapidly diversified from the late Pliocene (3.66 Ma) to the early Pleistocene (2.29 Ma), followed by a series of diversifications through the Pleistocene (Fig. 3). What factors are responsible for the rapid diversification of Chinese *Crocidura* distributed? We propose that climate change and rapid uplifting of the Qinghai-Tibet Plateau may have driven the diversification of Chinese *Crocidura*. Prevailing trends towards cooling and desiccation at the Pliocene/Pleistocene boundary [50] have led to the diversification of some taxa (e.g., [24, 26, 40]). Shrews are extremely sensitive to changes in ambient temperature and humidity, which are key factors affecting the distribution and number of shrews [51]. An arid environment will most likely serve as a barrier to their dispersal, and subsequent allopatric diversification of Chinese *Crocidura*. At the same time, global cooling induced habitat turnover and consequent further accelerated the diversification, and eventually lead to the formation of *Crocidura* species in the region [50, 52]. In addition, some geological studies also supported that rapid uplifting of the Qinghai-Tibet Plateau at 3.6 Ma, 1.8 Ma and 1.1 Ma have resulted in the diversifications of Chinese *Crocidura* distributed on the Tibetan plateau [53, 54].

### Taxonomic implications and diversity of *Crocidura* in China

Our data and analyses confirm the taxonomic status of the six well-recognized species: *C. shantungensis*, *C. dracula*, *C. suaveolens*, *C. vorax*, *C. lasiura*, and *C. tanakae* (Fig. 2; Additional file 2: Table S2). The mitochondrial and nuclear gene trees resolved each species as a well-demarcated clade, with substantial genetic differences between them (Table 3). Species delimitation analyses also identified each of them as a full species (Additional file 2: Table S2).

Jameson and Jones [55] originally described *C. horsfieldii kurodai*. Subsequently, Jiang and Hoffmann [2] revised the genus *Crocidura* in southern China and placed *C. tadae* and *C. kurodai* from Taiwan as junior synonyms of *C. rapax*. Hutterer [1] recognized three subspecies, *C. rapax tadae*, *C. r. kurodai*, and *C. r. lutaoensis* following Fang and Lee [19]. This view was followed by Hoffmann and Lunde [7], and Jenkins et al. [56]. Our analyses suggested the two subspecies, *C. rapax rapax* and *C. rapax kurodai*, should be elevated to full species status based on a high level of divergence (Table 3), GMYC and BPP analyses results (Fig. 3 and Additional file 2: Table S2).

Interestingly, when the *C. attenuata* samples from Vietnam were addedto the analysis, the phylogenetic tree showed that *C. r. rapax* was embedded within *C. attenuata*, making the latter a paraphyletic group (Fig. 3). Although the genetic distance value between these two species is only 0.049, this distance value is greater than the genetic distance value (= 0.039) between *C. kurodai* and *C. lasiura* (Table 3)*.* GMYC and BPP analysis strongly supported that they were two different species*.* We also examined a number of specimens including near type site specimens (Baoxing of Sichuan), and found that they were completely different in body size, skull size and altitude distribution (unpublished data). In addition, a *C. attenuata* population from Vietnam is supported as a separate species in GMYC. Therefore, a further research is needed to clarify the taxonomic status of the *C. attenuata* complex.

*Crocidura wuchihensis* was originally described in Hainan by Shou et al. [57]. Subsequently, Lunde et al. [58] identified one specimen from Vietnam as *C. wuchihensis*. Some specimens of *Crocidura* from Guangxi and Vietnam also have been referred to as *C. wuchihensis* [43]. Jenkins et al. [56] stated that *C. wuchihensis* was widely distributed across Vietnam, including the provinces Lao Cai, Ha Giang and Lang Son in the north and Ha Thinh and Quang Nam in Central Vietnam. Bannikova et al. [3] considered the distribution of *C. wuchihensis* to be restricted to areas east and north of the Red River. However, our analyses contradicted these assertions. Our analyses suggested that the population of *C. wuchihensis*_HN from Hainan and those of *C. wuchihensis*_GX from Guangxi and Vietnam are valid species, which implies that the true *C. wuchihensis* may only occur on Hainan Island. Our results call for the reappraisal of the taxonomic status of shrews previously referred to as *C. wuchihensis* from Guangxi and Vietnam.

*Crocidura indochinesis* were previously considered a subspecies of *C. horsfieldii* [59]. Subsequent, Lunde et al. [60] considered *C. indochinesis* from Ke Go Nature Reserve, Vietnam as a full species. Our GMYC analysis strongly supported it as a species. It is still unknown whether *C. indochinesis* is distributed in China, because we do not know if the population in southern China is the conspecific with those in Vietnam.

*Crocidura suaveolens* is not known in China [2]. However, Dubey et al. [61] and Bannikova et al. [62] considered *C. sibirica* distributed in Xinjiang province (Northwest China) as *Crocidura suaveolens*, based on multilocus phylogenetic data. Subsequently, specimens of *C.* aff*. Suaveolens* have been reported from southern Gansu (Northwest China) [63]. These specimens formed the sister-group of *C. suaveolens* in our phylogenic trees*.* Our GMYC analysis supported the population (*C.* aff*. suaveolens*) as a potential species (Fig. 3). In addition, Jiang and Hoffmann [2] listed *C. gmelini* from Central Asia, including Xinjiang in China. However, Ohdachi et al. [22] stated “It is possible that *C. gmelini* might be a synonym of *C. sibirica* (= *C. suaveolens*)”. All our *Crocidura* specimens from seven sample sites of Xinjiang in China appear to be *C. suaveolens* (Fig. 1). Therefore, it is necessary to sample *C. gmelini* to clarify the phylogenetic relationships and taxonomy between it and *C. suaveolens*. The occurrence of *C. gmelini* in China remains uncertain.

*Crocidura dracula* Thomas, 1912 [64] was first described from Mengzi County of Yunnan Province, China. Subsequently, the taxonomic decision was followed by Allen [4] and Ellerman and Morrison-Scott [65]. However, Jenkins [66] made *C. dracula* a subspecies of *C. fuliginosa.* Others have followed the arrangement [1, 2]. However, chromosomal and recently genetics studies suggested that *C. dracula* and *C. fuliginosa* are two different species [3, 8, 11, 29, 67]. Burgin and He [8] considered these two forms as full species. Our analyses also supported three clades of *C. fuliginosa* groups that represented three different species: *C. dracula* from Northern Vietnam and southern China, *C. fuliginosa* from southern Vietnam, Cambodia and Malaysia, and undescribed species (*C.* sp. 3) from Motuo of Xizang (West China). It is worth noting that the conspecificity of *C. fuliginosa* from Southern Vietnam, Cambodia and Malaysia with the type locality in Burma has still not been tested. In addition, a population was also reported as *C. dracula grisescens* in Zhejiang [68]. Jiang and Hoffmann [2] supposed that the population represents probably new taxon.

Our analyses also resolved another undescribed species, *Crocidura* sp. 1, despite only one specimen being available from Zada Coutry in Tibet. This species is closely related to the Zarudny’s rock shrew (*Crocidura zarudnyi*) from Iran based on the *cytb* gene. It is the sister-group of *C. shantungensis* and *C. suaveolens* based on nuclear genes. The condyloincisive length of the undescribed white-toothed shrew (19.55 mm; our unpublished data) is close to *C. attenuata* in China. Despite considerable effort, only one specimen was collected. Thus, our conclusions are therefore tentative. Additional fieldwork is needed to acquire new specimens and allow a comprehensive taxonomic and population genetic analysis. The continuous discovery of new species such as *Bufo zamdaensis* [69] and *Laudakia papenfussi* [70] shows a strong need for further exploration in the region.

Another undescribed species *C.* sp. 2 from Hongjiang County of Hunan and Dongyang County of Zhejiang was also strongly supported. It was not identified as any known species based on our morphological data. Genetically, it has a high level of divergence from all other members of the group (10.2–14.7%) (Table 3) and appears as a monophyletic group in the phylogenetic trees (Fig. 2; Fig. 3). Thus, the *C.* sp. 2 is likely another new species. Molecular analyses offer important insights, but extensive sampling, comprehensive morphological and morphometric comparisons are necessary to reach a final conclusion.

## Conclusions

In the present study, we obtained sequences of *Crocidura* throughout their distribution in China. We reconstructed the first multilocus phylogeny for the most speciose mammalian genus from China and found cryptic diversity. We propose that the three undescribed species should be evaluated using extensive taxon sampling and comprehensive morphological and morphometric approaches. Polyphyletic *C. wuchihensis* appears to be composed of two putative species. Two subspecies, *C. rapax rapax* and *C. rapax kurodai* should be elevated to full species status. Climate change since the late Pliocene periods and the uplift of the Qinghai-Tibet Plateau may have resulted in the diversification and speciation of China’s *Crocidura* species. In short, the underestimated diversity underlines the need for a taxonomic revision of Chinese *Crocidura* species.

## Methods

### Taxon sampling and data collection

A total of 117 *Crocidura* shrews from 49 localities in China were collected from 1997 to 2017 (Table 2; Fig. 1). Specimens were identified based on their morphology and distributions following Jiang and Hoffmann [2], and Hoffmann and Lunde [7]. If specimens were not able to be assigned to known species, they were assigned tentative into undescribed species. All field studies and lab work were approved by the Guidelines for Care and Use of Laboratory Animals and the Ethics Committee at Sichuan Normal University (Chengdu, China). These *Crocidura* shrews were caught throng Sherman trap, snap trap and pitfalls (plastic buckets that were 14 cm in diameter and 20 cm in depth), and then immediately euthanized by cervical dislocation. All efforts were made to minimize potential pain and suffering. Voucher specimens were deposited in the Nature Museum of the Sichuan Academy of Forestry and Sichuan Normal University. Muscle or liver tissue was collected and preserved in 95% ethanol and subsequently stored at − 80 °C for molecular studies.

To test phylogenetic relationship and divergence time estimation between Chinese *Crocidura* and the *Crocidura* shrews from Asia (including East Asia, South Asia and Southeast Asia), we also downloaded 214 *cytb* sequences from 70 species/ undescribed species available in GenBank for comparison (Additional file 3: Table S3). These taxa sampling also included 26 species including representatives of subfamily Myosoricinae (*Myosorex*), Soricinae (*Cryptotis*, *Otisorex*, *Anourosorex*, *Chodsigoa, Blarina*) and Crocidurinae (*Suncus*) as several external fossil calibration points for divergence time estimation (Additional file 3: Table S3). Divergence time trees were rooted by the sequences of *Uropsilus soricipes*. In adittion, Sequences of *Suncus murinus* of the subfamily Crocidurinae were chosen as outgroup for phylogenetic relationship inferring.

### DNA extraction and amplification

Total genomic DNA was extracted from muscle or liver using the phenol/proteinase K/sodium dodecyl sulfate method [71]. Given that the phylogenetic relationship of this *Crocidura* genus was well solved based on these four genes in the previous study [15, 29], we amplified these four genes, including the mitochondrial gene encoding cytochrome *b* (*cytb*) and nuclear gene fragments encoding Apolipoprotein B (*ApoB*), breast cancer susceptibility gene1 (*BRCA1*) and recombination activating gene 1 (*RAG1*).

Primer sets were taken from the literature (Additional file 4: Table S4). PCR amplifications were performed in a reaction volume mixture of 25 μl, containing 0.2 units of rTaq Polymerase (Takara, Dalian, China), 1× reaction buffer, 3 mM of MgCl_2_, 0.2 mM of each dNTP, 0.4 mM of each primer and approximately 100–500 ng of genomic DNA. PCR products were checked on a 1.0% agarose gel and purified using ethanol precipitation. Purified PCR products were directly sequenced using the BigDye terminator Cycle kit v3.1 (Applied Biosystems, Foster City, CA, USA) and determined with an ABI 310 analyzer (Applied Biosystems).

### Phylogenetic analyses and divergence time estimation

All DNA sequences were edited with EditSeq (DNASTAR, Lasergene v7.1) as well as aligned and examined by eye in MEGA 5 [72]. We applied Bayesian inference (BI) and maximum likelihood (ML) methods to infer the phylogenetic relationships. BI analyses were performed using BEAST v1.7.5 [73]. Analyses were conducted on the following four datasets: 1) a *cytb* gene dataset (mtDNA); 2) a three nuclear genes combined dataset (nDNA); 3) an all gene combined dataset (mtDNA + nDNA); and 4) the same as dataset 1 but with the *cytb* sequences of Asian *Crocidura* species and outgroups downloaded from GenBank (Additional file 3: Table S3). Each BEAST analysis used partition-specific models for the four genes (*cytb*, ApoB, BRCA1 and RAG1). The best model of evolution for each gene was determined using jModeltest v2 [74] ranked by the Akaike Information Criterion (AIC) (Additional file 1: Table S1). BEAST analyses used unlinked substitute model, linked clock models, linked tree, a random starting tree, a birth-death process tree prior, a relaxed lognormal clock model, and the program’s default prior distributions of model parameters.

We ran each analysis for 100 million generations, and sampled every 5000th generation. TRACER v1.6 [75] was used to confirm that the effective sample sizes (ESSs) were greater than 200 and the first 10% of the generations were treated as burn-in. Posterior probabilities (PP) > 0.95 were considered to be strongly supported [76]. ML analyses used RAxML v7.2.8 [77, 78] on the CIPRES Science Gateway v3.1 (http://www.phylo.org, [79]) and the GTRGAMMA model for each gene, as recommended. The analyses used the rapid bootstrapping algorithm [78] with 500 replicates.

Missing data will mislead estimates of branch lengths and affect the estimation of the divergence time [80]. We only used *cytb* (dataset 4) for the divergence time estimation for Asian *Crocidura* species because some species have no nuclear genes sequences available in GenBank (Additional file 3: Table S3). Due to the lack of a fossil record of this *Crocidura* group in Asia, a fossil calibration of a molecular clock was impossible. Following Jacquet et al. [81], we used five external calibration points derived from paleontological data of Soricomorpha to estimate times of divergence for the group. (1) The split between Soricinae and Crocidurinae – Myosoricinae is estimated to have occurred about 20 Ma [51] (normal: mean = 20 Ma, standard deviation = 1). (2) The oldest recorded Myosoricinae – Crocidurinae is dated to at least 12 Ma ago [82] (lognormal: mean = 0, stdve = 1, offset = 12 Ma). (3) The oldest known *Cryptotis* is dated to 9 Ma ago [83] (lognormal: mean = 0, stdve = 1, offset = 9 Ma). (4) The oldest *Otisorex* is dated to 3.5 Ma ago [84] (lognormal: mean = 0, stdve = 1, offset = 3.5 Ma). (5) The oldest *Crocidura* (*C. kapsominensis*) is dated to 6 Ma ago [85] (lognormal: mean = 0, stdve = 1, offset = 6 Ma).

Times of divergence and their credibility intervals within *Crocidura* were inferred using Bayesian analysis implemented with BEAST v1.7.5 [73]. BEAST analyses used unlinked substitute model, linked clock models, linked tree, a random starting tree, a birth-death process tree prior, a relaxed lognormal clock model, and the program’s default prior distributions of model parameters. Each analysis was run for 100 million generations and sampled every 5, 000 generations. The convergence of the run was checked by implementing TRACER v1.6 and the runs were combined using the Log combiner module of BEAST with a burn-in of 10%. All fossil calibration ages were treated as lognormal distributions [86] except the divergence between Soricinae and Crocidurinae–Myosoricinae, which was used as a constraint and thus treated as a normal distribution [30, 81, 87].

### Species delimitation and species tree

We first calculated the *p*-distances for *cytb* (dataset 1) between all species/ putative species using MEGA 5 [72]. We used the general mixed Yule-coalescent model (GMYC) [88, 89] methods to delineate species boundaries. The method is applicable for single-locus data. Our GMYC analyses were performed with mtDNA gene tree and the time-calibrated gene tree as the input tree derived from for dataset 1 and dataset 4 separately without outgroups, implemented in the R package SPILT [90].

We used Bayesian species-delimitation method to delineate species boundaries [91]. We tested the validity of our assignment of 14 putative species based on the results of SPLITS (see Results) using the guide-tree-free implemented in BPP v. 3.1 [91]. Only dataset 2 (nDNA) and dataset 3(mtDNA + nDNA) were included in the analysis. Both algorithms 0 and 1 were used to specify the rjMCMC moves between alternative models of species delimitation. Because primitively analyses showed that algorithms 0 and 1 produced similar results, algorithm 0 with fine tuning parameter was used for subsequent analysis. Gamma-distributed priors (G) were used to specify the ancestral population size (θ) and root age (τ). Use of inappropriate priors can bias posterior probabilities of species delimitation, potentially yielding false positives [91]. Thus, we tested two population size/tree age combinations chosen in previously studies [15, 92–94]. Trial runs showed good mixing using the two population size/tree age combinations. The two combinations were modeled to allow a range of speciation histories: shallow population size/ moderate divergence [G (2, 2000 forθand G (2, 2000) for τ] and large population size/ moderate divergence [G (1, 10) forθand G (2, 2000) for τ]. The divergence time prior (τ) used a diffuse Gamma-distributed probability distribution (2, 2000). The mean is 2/2000 = 0.001 (which means 0.1% of sequence divergence), which assumes that species split one million years ago if substitution rates are 2.2 × 10^− 9^ [95] and generation time is equal to 1 year. Each rjMCMC was run for 100,000 generations and sampled each 100 generations after discarding 10,000 generations as pre-burn-in.

The species tree was reconstructed for Chinese *Crocidura* based on a coalescent-based method implemented in *BEAST [96]. *Suncus murinus* was selected as outgroup. The analysis used the dataset 3 (mtDNA + nDNA). The best-fit models were calculated using jModeltest [74] and are provided in Additional file 1: Table S1. Samples were assigned to 15 putative species (including the outgroup species *Suncus murinus*) based on the results of SPLITS and BPP (see Results). We used the same priors as the phylogenetic analyses described above. Each analysis was run for 100 million generations and sampled every 5000th generation. Convergence of the run was checked using TRACER v1.6 and the runs were combined using the Log combiner module of BEAST with a burnin of 10%.

## Supplementary information

**Additional file 1:** **Table S1.** Haplotype diversity, nucleotide diversity and best substitution models for each gene used.

**Additional file 2:** **Table S2.** Posterior probabilities supporting undescribed species/species using different algorithms and priors.

**Additional file 3:** **Table S3.** Sampling information including localities and GenBank accession numbers for species used in this study.

**Additional file 4:** **Table S4.** Primers used for PCR and sequencing.

## Data Availability

The accession numbers for all genetic data used in this study are provided in Additional file 3: Table S3.
