# Supplementary material for: Multilocus phylogeny and cryptic diversity of white-toothed shrews (Mammalia, Eulipotyphla, Crocidura) in China
Source: BMC Evol Biol. 2020 Feb 14;20:29. doi: 10.1186/s12862-020-1588-8 (PMC7023792; doi:10.1186/s12862-020-1588-8)
Supplement: Supplementary file 1 — Additional file 1: Table S1. Haplotype diversity, nucleotide diversity and best substitution models for each gene used. [file 12862_2020_1588_MOESM1_ESM.doc]

**Table S1 Haplotype diversity，nucleotide diversity and best substitution models for each gene used**

| Partitions | Alignment | Haplotype diversity | Nucleotide diversity | Best Model |
| --- | --- | --- | --- | --- |
| *cytb* for dataset 1 | 1134bp | 0.985 | 0.11784 | GTR+I+G |
| APOB | 522bp | 0.956 | 0.02146 | GTR+G |
| BRCA1 | 793bp | 0.972 | 0.02141 | GTR+G |
| RAG1 | 855bp | 0.958 | 0.01366 | GTR+G |
| *cytb* for dataset 4 | 1134bp | - | - | GTR+I+G |
