# Supplementary material for: Multilocus phylogeny and cryptic diversity of white-toothed shrews (Mammalia, Eulipotyphla, Crocidura) in China
Source: BMC Evol Biol. 2020 Feb 14;20:29. doi: 10.1186/s12862-020-1588-8 (PMC7023792; doi:10.1186/s12862-020-1588-8)
Supplement: Supplementary file 3 — Additional file 3: Table S3. Sampling information including localities and GenBank accession numbers for species used in this study. [file 12862_2020_1588_MOESM3_ESM.docx]

**Table S3 Information of samples used in this study**

| **Species** | **Voucher ID** | **Sample localities** | ***cytb*** | **APOB** | **BRCA1** | **RAG1** | **Refrence** |
| --- | --- | --- | --- | --- | --- | --- | --- |
| *Crocidura* aff. *suaveolens* | GenBank | South Gansu, China | KX354172 | ﹣ | ﹣ | ﹣ | Sheftel, et al. 2018 |
| *Crocidura* aff. *suaveolens* | GenBank | South Gansu, China | KX354173 | ﹣ | ﹣ | ﹣ | Sheftel, et al. 2018 |
| *Crocidura* aff. *suaveolens* | GenBank | South Gansu, China | KX354174 | ﹣ | ﹣ | ﹣ | Sheftel, et al. 2018 |
| *Crocidura* aff. *suaveolens* | GenBank | South Gansu, China | KX354175 | ﹣ | ﹣ | ﹣ | Sheftel, et al. 2018 |
| *Crocidura* aff. *suaveolens* | GenBank | South Gansu, China | KX354177 | ﹣ | ﹣ | ﹣ | Sheftel, et al. 2018 |
| *Crocidura* aff. *suaveolens* | GenBank | South Gansu, China | KX354178 | ﹣ | ﹣ | ﹣ | Sheftel, et al. 2018 |
| *Crocidura* aff. *suaveolens* | GenBank | South Gansu, China | KX354180 | ﹣ | ﹣ | ﹣ | Sheftel, et al. 2018 |
| *Crocidura attenuata* | GenBank | Mt. Tay Con Linh II, Vietnam | AB175082 | ﹣ | ﹣ | ﹣ | Ohdachi, et al. 2004 |
| *Crocidura attenuata* | GenBank | Guangxi, China | EU122211 | ﹣ | ﹣ | ﹣ | Eesselstyn, et al. 2009 |
| *Crocidura attenuata* | GenBank | Ha Giang, Vietnam | GU358515 | ﹣ | ﹣ | ﹣ | Eesselstyn, et al. 2010 |
| *Crocidura attenuata* | GenBank | Coastal islands, Vietnam | JX181935 | ﹣ | ﹣ | ﹣ | Abramov, et al. 2012 |
| *Crocidura attenuata* | SQ01 | Qingchuan, Sichuan, China | MN690999 | ﹣ | ﹣ | ﹣ | in this study |
| *Crocidura attenuata* | SQ02 | Qingchuan, Sichuan, China | MN691000 | ﹣ | ﹣ | ﹣ | in this study |
| *Crocidura attenuata* | SQ04 | Qingchuan, Sichuan, China | MN691001 | ﹣ | ﹣ | ﹣ | in this study |
| *Crocidura attenuata* | SQ05 | Qingchuan, Sichuan, China | MN691002 | ﹣ | ﹣ | ﹣ | in this study |
| *Crocidura attenuata* | SQ06 | Pengzhou, Sichuan, China | MN691003 | ﹣ | ﹣ | ﹣ | in this study |
| *Crocidura attenuata* | SQ53 | Mingshan, Sichuan, China | MN691004 | MN690782 | MN690843 | MN690905 | in this study |
| *Crocidura attenuata* | SQ54 | Mingshan, Sichuan, China | MN691005 | MN690783 | MN690844 | MN690906 | in this study |
| *Crocidura attenuata* | SQ55 | Mingshan, Sichuan, China | MN691006 | MN690784 | MN690845 | MN690907 | in this study |
| *Crocidura attenuata* | SQ58 | Yingjing, Sichuan, China | MN691007 | MN690785 | MN690846 | MN690908 | in this study |
| *Crocidura attenuata* | SQ59 | Yingjing, Sichuan, China | MN691008 | MN690786 | MN690847 | MN690909 | in this study |
| *Crocidura attenuata* | SQ60 | Yingjing, Sichuan, China | MN691009 | MN690787 | MN690848 | MN690910 | in this study |
| *Crocidura attenuata* | SQ71 | Qingchuan, Sichuan, China | MN691010 | ﹣ | ﹣ | ﹣ | in this study |
| *Crocidura attenuata* | SQ72 | Yingjing, Sichuan, China | MN691011 | MN690788 | MN690849 | MN690911 | in this study |
| *Crocidura attenuata* | SQ80 | Qingchuan, Sichuan, China | MN691012 | ﹣ | ﹣ | ﹣ | in this study |
| *Crocidura attenuata* | SQ85 | Qingchuan, Sichuan, China | MN691013 | ﹣ | ﹣ | ﹣ | in this study |
| *Crocidura attenuata* | SQ89 | Muchuan, Sichuan, China | MN691014 | ﹣ | ﹣ | ﹣ | in this study |
| *Crocidura attenuata* | csd369 | Kaixian, Chongqing, China | MN691015 | ﹣ | ﹣ | ﹣ | in this study |
| *Crocidura attenuata* | csd468 | Huayingshan, Sichuan, China | MN691016 | ﹣ | ﹣ | ﹣ | in this study |
| *Crocidura batakorum* | GenBank | Palawan, Philippines | FJ813969 | ﹣ | ﹣ | ﹣ | Eesselstyn, et al. 2009 |
| *Crocidura batakorum* | GenBank | Palawan, Philippines | FJ813970 | ﹣ | ﹣ | ﹣ | Eesselstyn, et al. 2009 |
| *Crocidura batakorum* | GenBank | Palawan, Philippines | FJ813971 | ﹣ | ﹣ | ﹣ | Eesselstyn, et al. 2009 |
| *Crocidura beatus* | GenBank | Mindanao, Philippines | FJ813838 | ﹣ | ﹣ | ﹣ | Eesselstyn, et al. 2009 |
| *Crocidura beatus* | GenBank | Mindanao, Philippines | FJ813844 | ﹣ | ﹣ | ﹣ | Eesselstyn, et al. 2009 |
| *Crocidura beatus* | GenBank | Mindanao, Philippines | FJ813846 | ﹣ | ﹣ | ﹣ | Eesselstyn, et al. 2009 |
| *Crocidura beatus* | GenBank | Mt. Balatukan, Mindanao Island, Philippines | NC027249 | ﹣ | ﹣ | ﹣ | Giarla, et al. 2015 |
| *Crocidura beatus* | GenBank | Samar, Philippines | FJ814003 | ﹣ | ﹣ | ﹣ | Eesselstyn, et al. 2009 |
| *Crocidura beccarii* | GenBank | Mt. Singgalang, Sumatra, Indonesia | KX469561 | ﹣ | ﹣ | ﹣ | Demos, et al. 2016 |
| *Crocidura beccarii* | GenBank | Mt. Singgalang, Sumatra, Indonesia | KX469562 | ﹣ | ﹣ | ﹣ | Demos, et al. 2016 |
| *Crocidura beccarii* | GenBank | Mt. Singgalang, Sumatra, Indonesia | KX469563 | ﹣ | ﹣ | ﹣ | Demos, et al. 2016 |
| *Crocidura beccarii* | GenBank | Mt. Tujuh, Sumatra, Indonesia | KX469564 | ﹣ | ﹣ | ﹣ | Demos, et al. 2016 |
| *Crocidura beccarii* | GenBank | Mt. Tujuh, Sumatra, Indonesia | KX469566 | ﹣ | ﹣ | ﹣ | Demos, et al. 2016 |
| *Crocidura brunnea* | GenBank | Mt. Gede, Java, Indonesia | KF283220 | ﹣ | ﹣ | ﹣ | Eesselstyn, et al. 2013 |
| *Crocidura brunnea* | GenBank | Mt. Gede, Java, Indonesia | KF283221 | ﹣ | ﹣ | ﹣ | Eesselstyn, et al. 2013 |
| *Crocidura brunnea* | GenBank | Mt. Ciremai, Java, Indonesia | KF283249 | ﹣ | ﹣ | ﹣ | Eesselstyn, et al. 2013 |
| *Crocidura brunnea* | GenBank | Mt. Slamet, Java, Indonesia | KF283257 | ﹣ | ﹣ | ﹣ | Eesselstyn, et al. 2013 |
| *Crocidura brunnea* | GenBank | Mt. Slamet, Java, Indonesia | KF283259 | ﹣ | ﹣ | ﹣ | Eesselstyn, et al. 2013 |
| *Crocidura brunnea* | GenBank | Mt. Ijen, Java, Indonesia | KX469582 | ﹣ | ﹣ | ﹣ | Demos, et al. 2016 |
| *Crocidura brunnea* | GenBank | Mt. Salak, Java, Indonesia | KX469588 | ﹣ | ﹣ | ﹣ | Demos, et al. 2016 |
| *Crocidura* cf. *maxi* | GenBank | Flores, Indonesia | KF283278 | ﹣ | ﹣ | ﹣ | Eesselstyn, et al. 2013 |
| *Crocidura* cf. *maxi* | GenBank | Komodo, Indonesia | KF283277 | ﹣ | ﹣ | ﹣ | Eesselstyn, et al. 2013 |
| *Crocidura* cf. *monticola* | GenBank | Malay Peninsula | JX162650 | ﹣ | ﹣ | ﹣ | Omar, et al. 2013 |
| *Crocidura* cf. *monticola* | GenBank | Malay Peninsula | JX162656 | ﹣ | ﹣ | ﹣ | Omar, et al. 2013 |
| *Crocidura* cf. *monticola* | GenBank | Kalimantan, Indonesia | JX162666 | ﹣ | ﹣ | ﹣ | Omar, et al. 2013 |
| *Crocidura* cf. *monticola* | GenBank | Kalimantan, Indonesia | JX162667 | ﹣ | ﹣ | ﹣ | Omar, et al. 2013 |
| *Crocidura* cf. *monticola* | GenBank | Sabah, Malaysia | KF283275 | ﹣ | ﹣ | ﹣ | Eesselstyn, et al. 2013 |
| *Crocidura* cf. *tanakae* | GenBank | Tam Dao, Vietnam | FJ814021 | ﹣ | ﹣ | ﹣ | Eesselstyn, et al. 2009 |
| *Crocidura* cf. *tanakae* | GenBank | Tuyen Quang, Vietnam | FJ814031 | ﹣ | ﹣ | ﹣ | Eesselstyn, et al. 2009 |
| *Crocidura* cf. *tanakae* | GenBank | Quang Nam, Vietnam | FJ814032 | ﹣ | ﹣ | ﹣ | Eesselstyn, et al. 2009 |
| *Crocidura* cf. *tanakae* | GenBank | Ha Tinh, Vietnam | GU358520 | ﹣ | ﹣ | ﹣ | Eesselstyn, et al. 2010 |
| *Crocidura dracula* | GenBank | Yunnan, China | GU981271 | ﹣ | ﹣ | ﹣ | He, et al. 2010 |
| *Crocidura dracula* | GenBank | Taiwan, China | GU358519 | ﹣ | ﹣ | ﹣ | Eesselstyn, et al. 2010 |
| *Crocidura dracula* | GenBank | Ha Giang, Mt. Tay Con Linh II, Vietnam | AB175079 | ﹣ | ﹣ | ﹣ | Ohdachi, et al. 2004 |
| *Crocidura dracula* | SQ24 | Lijiang, Yunnan, China | MN690945 | MN690753 | MN690815 | MN690876 | in this study |
| *Crocidura dracula* | SQ28 | Lijiang, Yunnan, China | MN690946 | MN690754 | MN690816 | MN690877 | in this study |
| *Crocidura dracula* | SQ39 | Miyi, Sichuan, China | MN690947 | MN690755 | MN690817 | MN690878 | in this study |
| *Crocidura dracula* | SQ40 | Miyi, Sichuan, China | MN690948 | MN690756 | MN690818 | MN690879 | in this study |
| *Crocidura dracula* | SQ41 | Miyi, Sichuan, China | MN690949 | MN690757 | MN690819 | MN690880 | in this study |
| *Crocidura dracula* | SQ57 | Lijiang, Yunnan, China | MN690950 | MN690758 | MN690820 | MN690881 | in this study |
| *Crocidura dracula* | csd2388 | Yangbi, Yunnan, China | MN690951 | ﹣ | ﹣ | ﹣ | in this study |
| *Crocidura dracula* | csd2389 | Yangbi, Yunnan, China | MN690952 | ﹣ | ﹣ | ﹣ | in this study |
| *Crocidura dracula* | csd2390 | Yangbi, Yunnan, China | MN690953 | ﹣ | ﹣ | ﹣ | in this study |
| *Crocidura dsinezumi* | GenBank | Hokkaido, Japan | AB077059 | ﹣ | ﹣ | ﹣ | Ohdachi, et al. 2004 |
| *Crocidura dsinezumi* | GenBank | Hokkaido, Japan | AB077270 | ﹣ | ﹣ | ﹣ | Ohdachi, et al. 2004 |
| *Crocidura dsinezumi* | GenBank | Hokkaido, Japan | AB077276 | ﹣ | ﹣ | ﹣ | Ohdachi, et al. 2004 |
| *Crocidura elongata* | GenBank | Sulawesi, Indonesia | KX469590 | ﹣ | ﹣ | ﹣ | Demos, et al. 2016 |
| *Crocidura elongata* | GenBank | Sulawesi, Indonesia | KX469591 | ﹣ | ﹣ | ﹣ | Demos, et al. 2016 |
| *Crocidura elongata* | GenBank | Sulawesi, Indonesia | KY771756 | ﹣ | ﹣ | ﹣ | Eldridge, et al. 2018 |
| *Crocidura elongata* | GenBank | Sulawesi, Indonesia | KY771757 | ﹣ | ﹣ | ﹣ | Eldridge, et al. 2018 |
| *Crocidura elongata* | GenBank | Sulawesi, Indonesia | KY771758 | ﹣ | ﹣ | ﹣ | Eldridge, et al. 2018 |
| *Crocidura foetida* | GenBank | Sarawak, Malaysia | FJ814053 | ﹣ | ﹣ | ﹣ | Eesselstyn, et al. 2009 |
| *Crocidura foetida* | GenBank | Sarawak, Malaysia | FJ814054 | ﹣ | ﹣ | ﹣ | Eesselstyn, et al. 2009 |
| *Crocidura fuliginosa* | GenBank | Coastal islands, Vietnam | JX181941 | ﹣ | ﹣ | ﹣ | Abramov, et al. 2012 |
| *Crocidura fuliginosa* | GenBank | Pahang, Malaysia | FJ813925 | ﹣ | ﹣ | ﹣ | Eesselstyn, et al. 2009 |
| *Crocidura fuliginosa* | GenBank | Kampot, Cambodia | GU358522 | ﹣ | ﹣ | ﹣ | Eesselstyn, et al. 2010 |
| *Crocidura fuliginosa* | GenBank | Peninsular, Malaysia | FJ813924 | ﹣ | ﹣ | ﹣ | Eesselstyn, et al. 2009 |
| *Crocidura grayi* | GenBank | Luzon, Philippines | FJ813858 | ﹣ | ﹣ | ﹣ | Eesselstyn, et al. 2009 |
| *Crocidura grayi* | GenBank | Mindoro, Philippines | FJ813932 | ﹣ | ﹣ | ﹣ | Eesselstyn, et al. 2009 |
| *Crocidura grayi* | GenBank | Mindoro, Philippines | FJ813933 | ﹣ | ﹣ | ﹣ | Eesselstyn, et al. 2009 |
| *Crocidura grayi* | GenBank | Mindoro, Philippines | FJ813934 | ﹣ | ﹣ | ﹣ | Eesselstyn, et al. 2009 |
| *Crocidura grayi* | GenBank | Mindoro, Philippines | FJ813942 | ﹣ | ﹣ | ﹣ | Eesselstyn, et al. 2009 |
| *Crocidura grayi* | GenBank | Luzon, Philippines | FJ814049 | ﹣ | ﹣ | ﹣ | Eesselstyn, et al. 2009 |
| *Crocidura grayi* | GenBank | Luzon, Philippines | FJ813850 | ﹣ | ﹣ | ﹣ | Eesselstyn, et al. 2009 |
| *Crocidura grayi* | GenBank | Luzon, Philippines | FJ814048 | ﹣ | ﹣ | ﹣ | Eesselstyn, et al. 2009 |
| *Crocidura horsfieldii* | GenBank | Sri Lanka | EU122213 | ﹣ | ﹣ | ﹣ | Meegaskumbura, et al. 2007 |
| *Crocidura horsfieldii* | GenBank | Kanchana Buri, Sai Yok, Thailand | FJ814028 | ﹣ | ﹣ | ﹣ | Eesselstyn, et al. 2009 |
| *Crocidura indochinensis* | GenBank | Hon Giao, Vietnam | HM587023 | ﹣ | ﹣ | ﹣ | Bannlkova, et al. 2011 |
| *Crocidura indochinensis* | GenBank | Hon Giao, Vietnam | HM587024 | ﹣ | ﹣ | ﹣ | Bannlkova, et al. 2011 |
| *Crocidura lasiura* | GenBank | Ussuri, Russia | NC029329 | ﹣ | ﹣ | ﹣ | Kim, et al. 2017 |
| *Crocidura lasiura* | GenBank | Kraskino Vill, Primorye, Russia | AB077072 | ﹣ | ﹣ | ﹣ | Ohdachi, et al. 2004 |
| *Crocidura lasiura* | GenBank | Lazovsk Distric, tKhabarovsk Province, Russia | HM586997 | ﹣ | ﹣ | ﹣ | Bannlkova, et al. 2011 |
| *Crocidura lasiura* | GenBank | Imjin River, Korea | KJ004674 | ﹣ | ﹣ | ﹣ | Song, et al. 2009 |
| *Crocidura lasiura* | GenBank | Cixi, Zhejiang, China | KJ420558 | ﹣ | ﹣ | ﹣ | Lin, et al. 2014 |
| *Crocidura lasiura* | SQ99 | Xinbin, Liaoning, China | MN690957 | MN690762 | MN690824 | MN690885 | in this study |
| *Crocidura lasiura* | SQ100 | Xinbin, Liaoning, China | MN690958 | MN690763 | MN690825 | MN690886 | in this study |
| *Crocidura lasiura* | SQ101 | Xinbin, Liaoning, China | MN690959 | MN690764 | MN690826 | MN690887 | in this study |
| *Crocidura lasiura* | c132 | Fusong, Jilin, China | MN690960 | ﹣ | ﹣ | ﹣ | in this study |
| *Crocidura lasiura* | c133 | Fusong, Jilin, China | MN690961 | ﹣ | ﹣ | ﹣ | in this study |
| *Crocidura lasiura* | c134 | Fusong, Jilin, China | MN690962 | ﹣ | ﹣ | ﹣ | in this study |
| *Crocidura lepidura* | GenBank | Sumatra, Indonesia | FJ814022 | ﹣ | ﹣ | ﹣ | Eesselstyn, et al. 2009 |
| *Crocidura lepidura* | GenBank | Mt. Tujuh, Sumatra, Indonesia | KX469601 | ﹣ | ﹣ | ﹣ | Demos, et al. 2016 |
| *Crocidura lepidura* | GenBank | Mt. Tujuh, Sumatra, Indonesia | KX469602 | ﹣ | ﹣ | ﹣ | Demos, et al. 2016 |
| *Crocidura lepidura* | GenBank | Mt. Tujuh, Sumatra, Indonesia | KX469604 | ﹣ | ﹣ | ﹣ | Demos, et al. 2016 |
| *Crocidura lepidura* | GenBank | Sumatra, Indonesia | FJ814023 | ﹣ | ﹣ | ﹣ | Eesselstyn, et al. 2009 |
| *Crocidura malayana* | GenBank | Perak, Maxwell's Hill, Malaysia | DQ630381 | ﹣ | ﹣ | ﹣ | Dubey, et al. 2008 |
| *Crocidura maxi* | GenBank | Mt. Gede, Java, Indonesia | KF283195 | ﹣ | ﹣ | ﹣ | Eesselstyn, et al. 2013 |
| *Crocidura maxi* | GenBank | Mt. Gede, Java, Indonesia | KF283209 | ﹣ | ﹣ | ﹣ | Eesselstyn, et al. 2013 |
| *Crocidura maxi* | GenBank | Mt. Gede, Java, Indonesia | KF283233 | ﹣ | ﹣ | ﹣ | Eesselstyn, et al. 2013 |
| *Crocidura mindorus* | GenBank | Sibuyan, Philippines | FJ813841 | ﹣ | ﹣ | ﹣ | Eesselstyn, et al. 2009 |
| *Crocidura mindorus* | GenBank | Sibuyan, Philippines | FJ813842 | ﹣ | ﹣ | ﹣ | Eesselstyn, et al. 2009 |
| *Crocidura mindorus* | GenBank | Sibuyan, Philippines | FJ813843 | ﹣ | ﹣ | ﹣ | Eesselstyn, et al. 2009 |
| *Crocidura mindorus* | GenBank | Mindoro, Philippines | FJ813840 | ﹣ | ﹣ | ﹣ | Eesselstyn, et al. 2009 |
| *Crocidura miya* | GenBank | Agarapathana, Sri Lanka | EU122214 | ﹣ | ﹣ | ﹣ | Meegaskumbura, et al. 2007 |
| *Crocidura miya* | GenBank | Agarapathana, Sri Lanka | EU122215 | ﹣ | ﹣ | ﹣ | Meegaskumbura, et al. 2007 |
| *Crocidura miya* | GenBank | Agarapathana, Sri Lanka | EU122216 | ﹣ | ﹣ | ﹣ | Meegaskumbura, et al. 2007 |
| *Crocidura monticola* | GenBank | Mt. Gede, Java, Indonesia | KF283175 | ﹣ | ﹣ | ﹣ | Meegaskumbura, et al. 2007 |
| *Crocidura monticola* | GenBank | Mt. Salak, Java, Indonesia | KF283252 | ﹣ | ﹣ | ﹣ | Eesselstyn, et al. 2013 |
| *Crocidura monticola* | GenBank | Mt. Slamet, Java, Indonesia | KF283265 | ﹣ | ﹣ | ﹣ | Eesselstyn, et al. 2013 |
| *Crocidura monticola* | GenBank | Mt. Ijen, Java, Indonesia | KX469618 | ﹣ | ﹣ | ﹣ | Demos, et al. 2016 |
| *Crocidura musseri* | GenBank | Sulawesi, Indonesia | FJ813927 | ﹣ | ﹣ | ﹣ | Eesselstyn, et al. 2009 |
| *Crocidura neglecta* | GenBank | Sumatra, Indonesia | FJ814024 | ﹣ | ﹣ | ﹣ | Eesselstyn, et al. 2009 |
| *Crocidura neglecta* | GenBank | Mt. Tujuh, Sumatra, Indonesia | KX469628 | ﹣ | ﹣ | ﹣ | Demos, et al. 2016 |
| *Crocidura neglecta* | GenBank | Mt. Tujuh, Sumatra, Indonesia | KX469629 | ﹣ | ﹣ | ﹣ | Demos, et al. 2016 |
| *Crocidura negrina* | GenBank | Negros, Philippines | FJ813955 | ﹣ | ﹣ | ﹣ | Eesselstyn, et al. 2009 |
| *Crocidura negrina* | GenBank | Negros, Philippines | FJ813956 | ﹣ | ﹣ | ﹣ | Eesselstyn, et al. 2009 |
| *Crocidura negrina* | GenBank | Negros, Philippines | FJ813951 | ﹣ | ﹣ | ﹣ | Eesselstyn, et al. 2009 |
| *Crocidura negrina* | GenBank | Negros, Philippines | FJ813952 | ﹣ | ﹣ | ﹣ | Eesselstyn, et al. 2009 |
| *Crocidura nigripes* | GenBank | Sulavesi, Indonesia | DQ059024 | ﹣ | ﹣ | ﹣ | Dubey, et al. 2006 |
| *Crocidura nigripes* | GenBank | Sulawesi, Indonesia | FJ813926 | ﹣ | ﹣ | ﹣ | Eesselstyn, et al. 2009 |
| *Crocidura nigripes* | GenBank | Sulawesi, Indonesia | FJ813928 | ﹣ | ﹣ | ﹣ | Eesselstyn, et al. 2009 |
| *Crocidura orientalis* | GenBank | Mt. Gede, Java, Indonesia | KF283188 | ﹣ | ﹣ | ﹣ | Eesselstyn, et al. 2013 |
| *Crocidura orientalis* | GenBank | Mt. Ciremai, Java, Indonesia | KF283239 | ﹣ | ﹣ | ﹣ | Eesselstyn, et al. 2013 |
| *Crocidura orientalis* | GenBank | Mt. Ciremai, Java, Indonesia | KF283240 | ﹣ | ﹣ | ﹣ | Eesselstyn, et al. 2013 |
| *Crocidura orientalis* | GenBank | Mt. Ciremai, Java, Indonesia | KF283244 | ﹣ | ﹣ | ﹣ | Eesselstyn, et al. 2013 |
| *Crocidura orientalis* | GenBank | Mt. Slamet, Java, Indonesia | KF283262 | ﹣ | ﹣ | ﹣ | Eesselstyn, et al. 2013 |
| *Crocidura orientalis* | GenBank | Mt. Slamet, Java, Indonesia | KF283266 | ﹣ | ﹣ | ﹣ | Eesselstyn, et al. 2013 |
| *Crocidura orientalis* | GenBank | Mt. Slamet, Java, Indonesia | KF283268 | ﹣ | ﹣ | ﹣ | Eesselstyn, et al. 2013 |
| *Crocidura orii* | GenBank | Amami-ohshima Is, Kagoshima Pref, Japan | AB175087 | ﹣ | ﹣ | ﹣ | Ohdachi, et al. 2006 |
| *Crocidura palawanensis* | GenBank | Palawan, Philippines | FJ813902 | ﹣ | ﹣ | ﹣ | Eesselstyn, et al. 2009 |
| *Crocidura palawanensis* | GenBank | Palawan, Philippines | FJ813903 | ﹣ | ﹣ | ﹣ | Eesselstyn, et al. 2009 |
| *Crocidura palawanensis* | GenBank | Palawan, Philippines | FJ813918 | ﹣ | ﹣ | ﹣ | Eesselstyn, et al. 2009 |
| *Crocidura palawanensis* | GenBank | Palawan, Philippines | FJ813919 | ﹣ | ﹣ | ﹣ | Eesselstyn, et al. 2009 |
| *Crocidura panayensis* | GenBank | Panay, Philippines | FJ813944 | ﹣ | ﹣ | ﹣ | Eesselstyn, et al. 2009 |
| *Crocidura panayensis* | GenBank | Panay, Philippines | FJ813946 | ﹣ | ﹣ | ﹣ | Eesselstyn, et al. 2009 |
| *Crocidura panayensis* | GenBank | Panay, Philippines | FJ813945 | ﹣ | ﹣ | ﹣ | Eesselstyn, et al. 2009 |
| *Crocidura paradoxura* | GenBank | Mt. Singgalang, Sumatra, Indonesia | KX469632 | ﹣ | ﹣ | ﹣ | Demos, et al. 2016 |
| *Crocidura paradoxura* | GenBank | Mt. Singgalang, Sumatra, Indonesia | KX469633 | ﹣ | ﹣ | ﹣ | Demos, et al. 2016 |
| *Crocidura paradoxura* | GenBank | Mt. Tujuh, Sumatra, Indonesia | KX469640 | ﹣ | ﹣ | ﹣ | Demos, et al. 2016 |
| *Crocidura paradoxura* | GenBank | Mt. Tujuh, Sumatra, Indonesia | KX469641 | ﹣ | ﹣ | ﹣ | Demos, et al. 2016 |
| *Crocidura phanluongi* | GenBank | Yok Don National Park, Dak Lak Province, Vietnam | HM587020 | ﹣ | ﹣ | ﹣ | Bannlkova, et al. 2011 |
| *Crocidura phanluongi* | GenBank | coastal islands of Vietnam | JX181939 | ﹣ | ﹣ | ﹣ | Abramov, et al. 2012 |
| *Crocidura phuquocensis* | GenBank | Phu Quoc Isl, Kien Giang Province, Vietnam | HM587011 | ﹣ | ﹣ | ﹣ | Bannlkova, et al. 2011 |
| *Crocidura rapax kurodai* | GenBank | Nantou Xian, Xitou, Taiwan, China | AB057420 | ﹣ | ﹣ | ﹣ | Ohdachi, et al. 2001 |
| *Crocidura rapax kurodai* | GenBank | Nantou Xian, Xitou, Taiwan, China | AB062686 | ﹣ | ﹣ | ﹣ | Ohdachi, et al. 2001 |
| *Crocidura rapax kurodai* | GenBank | Taichung Hsien, Tahsueh Shan, Taiwan, China | AB115557 | ﹣ | ﹣ | ﹣ | Motokawa, et al. 2004 |
| *Crocidura rapax kurodai* | GenBank | Mt. Tay Con Linh II, Ha Giang, Vietnam | AB175086 | ﹣ | ﹣ | ﹣ | Ohdachi, et al. 2004 |
| *Crocidura rapax kurodai* | GenBank | Taiwan, China | GU358535 | ﹣ | ﹣ | ﹣ | Eesselstyn, et al. 2010 |
| *Crocidura rapax kurodai* | GenBank | Taiwan, China | GU358533 | ﹣ | ﹣ | ﹣ | Eesselstyn, et al. 2010 |
| *Crocidura rapax kurodai* | GenBank | Taiwan, China | GU358534 | ﹣ | ﹣ | ﹣ | Eesselstyn, et al. 2010 |
| *Crocidura rapax rapax* | SQ08 | Yajiang, Sichuan, China | MN690995 | MN690775 | MN690836 | MN690898 | in this study |
| *Crocidura rapax rapax* | SQ19 | Muli, Sichuan, China | MN690996 | MN690776 | MN690837 | MN690899 | in this study |
| *Crocidura rapax rapax* | SQ21 | Muli, Sichuan, China | MN690997 | MN690777 | MN690838 | MN690900 | in this study |
| *Crocidura rapax rapax* | SQ76 | Xiaojin, Sichuan, China | MN337427 | MN690778 | MN690839 | MN690901 | in this study |
| *Crocidura rapax rapax* | SQ77 | Xiaojin, Sichuan, China | MN337428 | MN690779 | MN690840 | MN690902 | in this study |
| *Crocidura rapax rapax* | SQ78 | Xiaojin, Sichuan, China | MN337429 | MN690780 | MN690841 | MN690903 | in this study |
| *Crocidura rapax rapax* | SQ94 | Jiulong, Sichuan, China | MN690998 | MN690781 | MN690842 | MN690904 | in this study |
| *Crocidura shantungensis* | GenBank | West Khingan, Mongolia | EU742589 | ﹣ | ﹣ | ﹣ | Bannikova, et al. 2009 |
| *Crocidura shantungensis* | GenBank | Taiwan, China | FJ814020 | ﹣ | ﹣ | ﹣ | Eesselstyn, et al. 2009 |
| *Crocidura shantungensis* | GenBank | Jeju Island, Korea | HQ709234 | ﹣ | ﹣ | ﹣ | Arai, et al. 2012 |
| *Crocidura shantungensis* | GenBank | Busan, Korea | KF144152 | ﹣ | ﹣ | ﹣ | Koh, et al. 2013 |
| *Crocidura shantungensis* | SQ46 | Ningwu, Shanxi, China | MN690925 | MN690740 | MN690802 | MN690863 | in this study |
| *Crocidura shantungensis* | SQ93 | Zunhua, Hebei, China | MN690926 | MN690741 | MN690803 | MN690864 | in this study |
| *Crocidura shantungensis* | SQ96 | Xinbin, Liaoning, China | MN690927 | MN690742 | MN690804 | MN690865 | in this study |
| *Crocidura shantungensis* | SQ97 | Xinbin, Liaoning, China | MN690928 | MN690743 | MN690805 | MN690866 | in this study |
| *Crocidura shantungensis* | SQ98 | Xinbin, Liaoning, China | MN690929 | MN690744 | MN690806 | MN690867 | in this study |
| *Crocidura sokolovi* | GenBank | Mt. Ngoc Linh, Kon Tum Province, Vietnam | HM586998 | ﹣ | ﹣ | ﹣ | Bannlkova, et al. 2011 |
| *Crocidura sokolovi* | GenBank | Mt. Ngoc Linh, Kon Tum Province, Vietnam | HM586999 | ﹣ | ﹣ | ﹣ | Bannlkova, et al. 2011 |
| *Crocidura* sp. 1 | Zada | Zada, Xizang, China | MN691017 | MN690789 | MN690850 | MN690912 | in this study |
| *Crocidura* sp. 1_2009 | GenBank | Sulawesi, Indonesia | FJ814025 | ﹣ | ﹣ | ﹣ | Eesselstyn, et al. 2009 |
| *Crocidura* sp. 1_2013 | GenBank | Mt. Gede, Java, Indonesia | KF283204 | ﹣ | ﹣ | ﹣ | Eesselstyn, et al. 2013 |
| *Crocidura* sp. 2 | csd095 | Hongjiang, Hunan, China | MN691018 | MN690790 | MN690851 | MN690913 | in this study |
| *Crocidura* sp. 2 | csd098 | Hongjiang, Hunan, China | MN691019 | MN690791 | MN690852 | MN690914 | in this study |
| *Crocidura* sp. 2 | csd1017 | Dongyang, Zhejiang, China | MN691020 | ﹣ | ﹣ | ﹣ | in this study |
| *Crocidura* sp. 2 | csd1019 | Dongyang, Zhejiang, China | MN691021 | ﹣ | ﹣ | ﹣ | in this study |
| *Crocidura* sp. 2 | csd1021 | Dongyang, Zhejiang, China | MN691022 | ﹣ | ﹣ | ﹣ | in this study |
| *Crocidura* sp. 2 | csd1022 | Dongyang, Zhejiang, China | MN691023 | ﹣ | ﹣ | ﹣ | in this study |
| *Crocidura* sp. 2 | csd1025 | Dongyang, Zhejiang, China | MN691024 | ﹣ | ﹣ | ﹣ | in this study |
| *Crocidura* sp. 2 | csd1026 | Dongyang, Zhejiang, China | MN691025 | MN690792 | MN690853 | MN690915 | in this study |
| *Crocidura* sp. 2 | csd1030 | Dongyang, Zhejiang, China | MN691026 | ﹣ | ﹣ | ﹣ | in this study |
| *Crocidura* sp. 2 | csd1036 | Dongyang, Zhejiang, China | MN691027 | MN690793 | MN690854 | MN690916 | in this study |
| *Crocidura* sp. 2 | GenBank | Sulawesi, Indonesia | FJ814026 | ﹣ | ﹣ | ﹣ | Eesselstyn, et al. 2009 |
| *Crocidura* sp. 3 | GenBank | Sulawesi, Indonesia | FJ814027 | ﹣ | ﹣ | ﹣ | Eesselstyn, et al. 2009 |
| *Crocidura* sp. 3 | SQ31 | Motuo, Xizang, China | MN691028 | MN690794 | MN690855 | MN690917 | in this study |
| *Crocidura* sp. 3 | SQ32 | Motuo, Xizang, China | MN691029 | MN690795 | MN690856 | MN690918 | in this study |
| *Crocidura* sp. 3 | SQ33 | Motuo, Xizang, China | MN691030 | MN690796 | MN690857 | MN690919 | in this study |
| *Crocidura* sp. 3 | SQ84 | Motuo, Xizang, China | MN691031 | MN690797 | MN690858 | MN690920 | in this study |
| *Crocidura* sp. 3 | csd874 | Motuo, Xizang, China | MN691032 | ﹣ | ﹣ | ﹣ | in this study |
| *Crocidura* sp. 4 | GenBank | Cambodia | GU358523 | ﹣ | ﹣ | ﹣ | Eesselstyn, et al. 2010 |
| *Crocidura* sp. AB1 | GenBank | Lao Cai Province, Sa Pa, Vietnam | HM587006 | ﹣ | ﹣ | ﹣ | Bannlkova, et al. 2011 |
| *Crocidura* sp. AB2 | GenBank | Binh Chau - Phuoc Buu Nature, Ba Ria Vung Tau Province, Vietnam | HM587018 | ﹣ | ﹣ | ﹣ | Bannlkova, et al. 2011 |
| *Crocidura* sp. AB2 | GenBank | Binh Chau, Vietnam | JX181937 | ﹣ | ﹣ | ﹣ | Abramov, et al. 2012 |
| *Crocidura* sp. AB2 | GenBank | Binh Chau, Vietnam | JX181938 | ﹣ | ﹣ | ﹣ | Abramov, et al. 2012 |
| *Crocidura* sp. nov. 1 | GenBank | Mt. Gede, Java, Indonesia | KF283189 | ﹣ | ﹣ | ﹣ | Eesselstyn, et al. 2013 |
| *Crocidura* sp. nov. 1 | GenBank | Mt. Gede, Java, Indonesia | KF283207 | ﹣ | ﹣ | ﹣ | Eesselstyn, et al. 2013 |
| *Crocidura* sp. nov. 2 | GenBank | Mt. Singgalang, Sumatra, Indonesia | KX469648 | ﹣ | ﹣ | ﹣ | Demos, et al. 2016 |
| *Crocidura* sp. nov. 2 | GenBank | Mt. Singgalang, Sumatra, Indonesia | KX469649 | ﹣ | ﹣ | ﹣ | Demos, et al. 2016 |
| *Crocidura* sp. nov. 2 | GenBank | Mt. Singgalang, Sumatra, Indonesia | KX469650 | ﹣ | ﹣ | ﹣ | Demos, et al. 2016 |
| *Crocidura suaveolens* | GenBank | Xinjiang Province, China | AB077083 | ﹣ | ﹣ | ﹣ | Ohdachi, et al. 2004 |
| *Crocidura suaveolens* | GenBank | Sharga Vill, Mongolia | AB077087 | ﹣ | ﹣ | ﹣ | Ohdachi, et al. 2004 |
| *Crocidura suaveolens* | GenBank | Central Siberia, Sharg vil, Mongolia | AB077088 | ﹣ | ﹣ | ﹣ | Ohdachi, et al. 2004 |
| *Crocidura suaveolens* | GenBank | Buryatian, Russia | EU742583 | ﹣ | ﹣ | ﹣ | Bannikova, et al. 2009 |
| *Crocidura suaveolens* | GenBank | Teletzkoe Lake, Russia | HM586993 | ﹣ | ﹣ | ﹣ | Bannlkova, et al. 2011 |
| *Crocidura suaveolens* | GenBank | Teletzkoe Lake, Russia | HM586995 | ﹣ | ﹣ | ﹣ | Bannlkova, et al. 2011 |
| *Crocidura suaveolens* | SQ64 | Aletai, Xinjiang, China | MN690930 | MN690745 | MN690807 | MN690868 | in this study |
| *Crocidura suaveolens* | SQ65 | Aletai, Xinjiang, China | MN690931 | MN690746 | MN690808 | MN690869 | in this study |
| *Crocidura suaveolens* | SQ66 | Aletai, Xinjiang, China | MN690932 | MN690747 | MN690809 | MN690870 | in this study |
| *Crocidura suaveolens* | SQ67 | Aletai, Xinjiang, China | MN690933 | MN690748 | MN690810 | MN690871 | in this study |
| *Crocidura suaveolens* | SQ68 | Aletai, Xinjiang, China | MN690934 | MN690749 | MN690811 | MN690872 | in this study |
| *Crocidura suaveolens* | SQ69 | Aletai, Xinjiang, China | MN690935 | MN690750 | MN690812 | MN690873 | in this study |
| *Crocidura suaveolens* | SQ70 | Aletai, Xinjiang, China | MN690936 | MN690751 | MN690813 | MN690874 | in this study |
| *Crocidura suaveolens* | SQ88 | Aletai, Xinjiang, China | MN690937 | MN690752 | MN690814 | MN690875 | in this study |
| *Crocidura suaveolens* | csd2343 | Tashenkuergan, Xinjiang, China | MN690938 | ﹣ | ﹣ | ﹣ | in this study |
| *Crocidura suaveolens* | csd2344 | Tashenkuergan, Xinjiang, China | MN690939 | ﹣ | ﹣ | ﹣ | in this study |
| *Crocidura suaveolens* | csd2345 | Xinjiang, China | MN690940 | ﹣ | ﹣ | ﹣ | in this study |
| *Crocidura suaveolens* | csd2346 | Xinjiang, China | MN690941 | ﹣ | ﹣ | ﹣ | in this study |
| *Crocidura suaveolens* | csd2347 | Xinjiang, China | MN690942 | ﹣ | ﹣ | ﹣ | in this study |
| *Crocidura suaveolens* | csd2348 | Xinjiang, China | MN690943 | ﹣ | ﹣ | ﹣ | in this study |
| *Crocidura suaveolens* | csd2349 | Xinjiang, China | MN690944 | ﹣ | ﹣ | ﹣ | in this study |
| *Crocidura tanakae* | GenBank | Taiwan, Nantou, China | AB175080 | ﹣ | ﹣ | ﹣ | Ohdachi, et al. 2004 |
| *Crocidura tanakae* | GenBank | Taiwan, Nantou, China | AB175081 | ﹣ | ﹣ | ﹣ | Ohdachi, et al. 2004 |
| *Crocidura tanakae* | GenBank | Taiwan, China | GU358530 | ﹣ | ﹣ | ﹣ | Eesselstyn, et al. 2010 |
| *Crocidura tanakae* | GenBank | Taiwan, China | GU358531 | ﹣ | ﹣ | ﹣ | Eesselstyn, et al. 2010 |
| *Crocidura tanakae* | GenBank | Batanes, Philippines | GU358538 | ﹣ | ﹣ | ﹣ | Eesselstyn, et al. 2010 |
| *Crocidura tanakae* | GenBank | Mt Bi Doup, Lam Dong Province, Vietnam | HM587027 | ﹣ | ﹣ | ﹣ | Bannlkova, et al. 2011 |
| *Crocidura tanakae* | GenBank | Ban Doy Area, Khammouane Province, Laos | HM587031 | ﹣ | ﹣ | ﹣ | Bannlkova, et al. 2011 |
| *Crocidura tanakae* | GenBank | Mt. Tay Con Linh II, Vietnam | AB175083 | ﹣ | ﹣ | ﹣ | Ohdachi, et al. 2004 |
| *Crocidura tanakae* | SQ43 | Mt. Fanjingshan, Guizhou, China | MN690963 | MN690765 | MN690827 | MN690888 | in this study |
| *Crocidura tanakae* | SQ47 | Mt. Fanjingshan, Guizhou, China | MN690964 | ﹣ | ﹣ | ﹣ | in this study |
| *Crocidura tanakae* | SQ56 | Chongzuo, Guangxi, China | MN690965 | ﹣ | ﹣ | ﹣ | in this study |
| *Crocidura tanakae* | SQ90 | Langzhong, Sichuan, China | MN690966 | MN690766 | MN690828 | MN690889 | in this study |
| *Crocidura tanakae* | SQ91 | Chongzuo, Guangxi, China | MN690967 | ﹣ | ﹣ | ﹣ | in this study |
| *Crocidura tanakae* | SQ92 | Chongzuo, Guangxi, China | MN690968 | ﹣ | ﹣ | ﹣ | in this study |
| *Crocidura tanakae* | csd092 | Hongjiang, Hunan, China | MN690969 | ﹣ | ﹣ | ﹣ | in this study |
| *Crocidura tanakae* | csd094 | Hongjiang, Hunan, China | MN690970 | ﹣ | ﹣ | ﹣ | in this study |
| *Crocidura tanakae* | csd097 | Hongjiang, Hunan, China | MN690971 | ﹣ | ﹣ | ﹣ | in this study |
| *Crocidura tanakae* | csd223 | Yuelushan, Hunan, China | MN690972 | ﹣ | ﹣ | ﹣ | in this study |
| *Crocidura tanakae* | csd224 | Hengshan, Hunan, China | MN690973 | ﹣ | ﹣ | ﹣ | in this study |
| *Crocidura tanakae* | csd225 | Yuelushan, Hunan, China | MN690974 | ﹣ | ﹣ | ﹣ | in this study |
| *Crocidura tanakae* | csd226 | Hengshan, Hunan, China | MN690975 | ﹣ | ﹣ | ﹣ | in this study |
| *Crocidura tanakae* | csd227 | Hengshan, Hunan, China | MN690976 | ﹣ | ﹣ | ﹣ | in this study |
| *Crocidura tanakae* | csd338 | Hejiang, Sichuan, China | MN690977 | MN690767 | MN690829 | MN690890 | in this study |
| *Crocidura tanakae* | csd371 | Hejiang, Sichuan, China | MN690978 | MN690768 | MN690830 | MN690891 | in this study |
| *Crocidura tanakae* | csd398 | Yuelushan, Hunan, China | MN690979 | ﹣ | ﹣ | ﹣ | in this study |
| *Crocidura tanakae* | csd466 | Huayingshan, Sichuan, China | MN690980 | ﹣ | ﹣ | ﹣ | in this study |
| *Crocidura tanakae* | csd467 | Huayingshan, Sichuan, China | MN690981 | ﹣ | ﹣ | ﹣ | in this study |
| *Crocidura tanakae* | csd469 | Huayingshan, Sichuan, China | MN690982 | ﹣ | ﹣ | ﹣ | in this study |
| *Crocidura tanakae* | csd907 | Santai, Sichuan, China | MN690983 | ﹣ | ﹣ | ﹣ | in this study |
| *Crocidura tanakae* | csd1023 | Dongyang, Zhejiang, China | MN690984 | ﹣ | ﹣ | ﹣ | in this study |
| *Crocidura tanakae* | csd1024 | Dongyang, Zhejiang, China | MN690985 | ﹣ | ﹣ | ﹣ | in this study |
| *Crocidura tanakae* | csd1027 | Dongyang, Zhejiang, China | MN690986 | ﹣ | ﹣ | ﹣ | in this study |
| *Crocidura tanakae* | csd1028 | Dongyang, Zhejiang, China | MN690987 | ﹣ | ﹣ | ﹣ | in this study |
| *Crocidura tanakae* | csd1029 | Dongyang, Zhejiang, China | MN690988 | ﹣ | ﹣ | ﹣ | in this study |
| *Crocidura tanakae* | djy018 | Dujiangyan, Sichuan, China | MN690989 | MN690769 | MN690831 | MN690892 | in this study |
| *Crocidura tanakae* | djy027 | Dujiangyan, Sichuan, China | MN690990 | ﹣ | ﹣ | ﹣ | in this study |
| *Crocidura tanakae* | EMEI | Emei, Sichuan, China | MN690991 | ﹣ | ﹣ | ﹣ | in this study |
| *Crocidura tanakae* | GZSQ | Shiqian, Guizhou, China | MN690992 | MN690770 | MN690832 | MN690893 | in this study |
| *Crocidura tanakae* | 14N145 | Mt. Emei, Sichuan, China | MN690993 | MN690771 | MN690833 | MN690894 | in this study |
| *Crocidura vorax* | SQ22 | Mt. Jinfo, Chongqing, China | MN690994 | MN690772 | MN690834 | MN690895 | in this study |
| *Crocidura vorax* | SQ44 | Songtao, Guizhou, China | MN337431 | MN690773 | MN690835 | MN690896 | in this study |
| *Crocidura vorax* | csd1378 | Mt. Leigongshan, Guizhou, China | MN337430 | MN690774 | ﹣ | MN690897 | in this study |
| *Crocidura vosmaeri* | GenBank | Bangka Island, Sumatra, Indonesia | KX469663 | ﹣ | ﹣ | ﹣ | Demos, et al. 2016 |
| *Crocidura vosmaeri* | GenBank | Bangka Island, Sumatra, Indonesia | KX469664 | ﹣ | ﹣ | ﹣ | Demos, et al. 2016 |
| *Crocidura vosmaeri* | GenBank | Bangka Island, Sumatra, Indonesia | KX469665 | ﹣ | ﹣ | ﹣ | Demos, et al. 2016 |
| *Crocidura watasei* | GenBank | Tokunoshima, Japan | AB077074 | ﹣ | ﹣ | ﹣ | Ohdachi, et al. 2004 |
| *Crocidura wuchihensis*_GX | GenBank | Guangxi Province, China | FJ814041 | ﹣ | ﹣ | ﹣ | Eesselstyn, et al. 2009 |
| *Crocidura wuchihensis*_GX | GenBank | Ha Giang, Mt. Tay Con Linh II, Vietnam | AB175084 | ﹣ | ﹣ | ﹣ | Ohdachi, et al. 2004 |
| *Crocidura wuchihensis*_GX | GenBank | Ha Giang, Mt. Tay Con Linh II, Vietnam | AB175085 | ﹣ | ﹣ | ﹣ | Ohdachi, et al. 2004 |
| *Crocidura wuchihensis*_GX | GenBank | Guangxi Province, China | FJ814040 | ﹣ | ﹣ | ﹣ | Eesselstyn, et al. 2009 |
| *Crocidura wuchihensis*_HN | SQ11 | Mt. Diaoluo, Hainan, China | MN690954 | MN690759 | MN690821 | MN690882 | in this study |
| *Crocidura wuchihensis*_HN | SQ12 | Mt. Diaoluo, Hainan, China | MN690955 | MN690760 | MN690822 | MN690883 | in this study |
| *Crocidura wuchihensis*_HN | SQ13 | Mt. Diaoluo, Hainan, China | MN690956 | MN690761 | MN690823 | MN690884 | in this study |
| *Crocidura zaitsevi* | GenBank | Kon Tum Province, Vietnam | HM587002 | ﹣ | ﹣ | ﹣ | Bannlkova, et al. 2011 |
| *Crocidura zaitsevi* | GenBank | Kon Tum Province, Vietnam | HM587004 | ﹣ | ﹣ | ﹣ | Bannlkova, et al. 2011 |
| *Crocidura zaitsevi* | GenBank | Lam Dong Province, Vietnam | HM587021 | ﹣ | ﹣ | ﹣ | Bannlkova, et al. 2011 |
| *Crocidura zaitsevi* | GenBank | Hon Giao, Vietnam | HM587022 | ﹣ | ﹣ | ﹣ | Bannlkova, et al. 2011 |
| *Crocidura zaitsevi* | GenBank | Lam Dong Province, Vietnam | HM587026 | ﹣ | ﹣ | ﹣ | Bannlkova, et al. 2011 |
| *Crocidura zarudnyi* | GenBank | Baluchestan, Iran | AY925211 | ﹣ | ﹣ | ﹣ | Dubey, et al. 2007 |
| *Anourosorex squamipes* | GenBank | Doi Inthanon, Thailand | AB175091 | ﹣ | ﹣ | ﹣ | Ohdachi, et al. 1997 |
| *Blarina brevicauda* | GenBank | Michigan State, America | DQ630416 | ﹣ | ﹣ | ﹣ | Dubey, et al. 2007 |
| *Blarinella griselda* | GenBank | Ha Giang; Mt. Tay Con Linh II, Vietnam | AB175144 | ﹣ | ﹣ | ﹣ | Ohdachi, et al. 1997 |
| *Chodsigoa parca* | GenBank | Yunnan, China | GU981265 | ﹣ | ﹣ | ﹣ | He, et al. 2010 |
| *Cryptotis goldmani* | GenBank | Omiltemi, Gurrero, Mexico | AB175138 | ﹣ | ﹣ | ﹣ | Ohdachi, et al. 1997 |
| *Cryptotis magna* | GenBank | Llano de las Flores, Oaxaca, Mexico | AB175141 | ﹣ | ﹣ | ﹣ | Ohdachi, et al. 1997 |
| *Cryptotis parva* | GenBank | Tom Green County, Texas, America | AB175135 | ﹣ | ﹣ | ﹣ | Ohdachi, et al. 1997 |
| *Diplomesodon pulchellum* | GenBank | Palawan Island, Philippines | MG973433 | ﹣ | ﹣ | ﹣ | Hutterer, et al. 2018 |
| *Episoriculus leucops* | GenBank | Yunnan Province, China | GU981284 | ﹣ | ﹣ | ﹣ | He, et al. 2010 |
| *Myosorex cafer* | GenBank | Serala Province. Nature Res, South Africa | DQ630418 | ﹣ | ﹣ | ﹣ | Dubey, et al. 2007 |
| *Myosorex sclateri* | GenBank | Natal, Matubatuba, South Africa | DQ630436 | ﹣ | ﹣ | ﹣ | Dubey, et al. 2007 |
| *Nectogale elegans* | GenBank | Yunnan Province, China | GU981294 | ﹣ | ﹣ | ﹣ | He, et al. 2010 |
| *Neomys fodiens* | GenBank | Heilongjiang Province, China | NC025559 | ﹣ | ﹣ | ﹣ | Liu, et al. 2016 |
| *Sorex bedfordiae* | GenBank | Yunnan Province, China | GU981296 | ﹣ | ﹣ | ﹣ | He, et al. 2010 |
| *Sorex cinereus* | GenBank | Pennsylvania, Westmoreland County, America | AY014952 | ﹣ | ﹣ | ﹣ | Cook, et al. 2003 |
| *Sorex fumeus* | GenBank | Pennsylvania State, America | AB175116 | ﹣ | ﹣ | ﹣ | Ohdachi, et al. 1997 |
| *Sorex fumeus* | GenBank | Pennsylvania State, America | DQ630414 | ﹣ | ﹣ | ﹣ | Dubey, et al. 2007 |
| *Sorex saussurei* | GenBank | Guerrero State, Mexico | AB175118 | ﹣ | ﹣ | ﹣ | Ohdachi, et al. 1997 |
| *Soriculus nigrescens* | GenBank | Yunnan Province, China | GU981301 | ﹣ | ﹣ | ﹣ | He, et al. 2010 |
| *Suncus etruscus* | GenBank | Anuradhapura, Sri Lanka | FJ716836 | ﹣ | ﹣ | ﹣ | Meegaskumbura, et al. 2009 |
| *Suncus etruscus* | GenBank | South India | JN556043 | ﹣ | ﹣ | ﹣ | Meegaskumbura, et al. 2012 |
| *Suncus etruscus* | GenBank | Khuzestan state, Haffar-e Shargi, Iran | LC126597 | ﹣ | ﹣ | ﹣ | Ohdachi, et al. 2016 |
| *Suncus murinus* | GenBank | Taichung, Taiwan, China | AB175075 | ﹣ | ﹣ | ﹣ | Ohdachi, et al. 2004 |
| *Suncus murinus* | GenBank | Anuradhapura, Sri Lanka | EU122224 | ﹣ | ﹣ | ﹣ | Meegaskumbura, et al. 2007 |
| *Suncus murinus* | GenBank | Pokunuthanne, Udawalawe, Sri Lanka | GQ290380 | ﹣ | ﹣ | ﹣ | Meegaskumbura, et al. 2010 |
| *Suncus murinus* | GenBank | Wencheng, Zhejiang Province, China | KJ420549 | ﹣ | ﹣ | ﹣ | Lin, et al. 2014 |
| *Suncus murinus* | GenBank | Yuhua, Zhejiang Province, China, | KJ420555 | ﹣ | ﹣ | ﹣ | Lin, et al. 2014 |
| *Suncus murinus* | GenBank | Yulin Normal University of Yulin, Guangxi Province, China | NC024604 | ﹣ | ﹣ | ﹣ | Chen, et al. 2016 |
| *Suncus murinus* | chou2 | Yulin, Guangxi, China | MN691033 | MN690798 | MN690859 | MN690921 | in this study |
| *Suncus murinus* | chou3 | Yulin, Guangxi, China | MN691034 | MN690799 | MN690860 | MN690922 | in this study |
| *Suncus murinus* | chou4 | Yulin, Guangxi, China | MN691035 | MN690800 | MN690861 | MN690923 | in this study |
| *Suncus murinus* | chou5 | Yulin, Guangxi, China | MN691036 | MN690801 | MN690862 | MN690924 | in this study |
| *Uropsilus soricipes* | GenBank | Mt. Jiajin, Sichuan Province, China | JQ695828 | ﹣ | ﹣ | ﹣ | Tu, et al. 2014 |
| *Uropsilus soricipes* | GenBank | Mt. Jiajin, Sichuan Province, China | JQ695829 | ﹣ | ﹣ | ﹣ | Tu, et al. 2014 |

**Refrence**

Abramov, A. V., Bannikova, A. A., Rozhnov, V. V. (2012). White-toothed shrews (Mammalia, Soricomorpha, *Crocidura*) of coastal islands of Vietnam. ZooKeys, (207), 37.

Arai, S., Gu, S. H., Baek, L. J., Tabara, K., Bennett, S. N., Oh, H. S., Okabe, N. (2012). Divergent ancestral lineages of newfound hantaviruses harbored by phylogenetically related Crocidurine shrew species in Korea. Virology, 424(2), 99-105.

Bannikova, A. A., Abramov, A. V., Borisenko, A. V., Lebedev, V. S., Rozhnov, V. V. (2011). Mitochondrial diversity of the white-toothed shrews (Mammalia, Eulipotyphla, *Crocidura*) in Vietnam. Zootaxa, 2812, 1-20.

Chen, S., Wei, H., Peng, H., Yong, B. (2016). The complete mitogenome of Asian house shrews, *Suncus murinus* (Soricidae). Mitochondrial DNA Part A, 27(2), 1127-1128.

Cook, D. J. A. (2003). Phylogenetic diversification within the *Sorex cinereus* group (Soricidae). Journal of Mammalogy, 84(1), 144-158.

Demos, T. C., Achmadi, A. S., Giarla, T. C., Handika, H., Rowe, K. C., Esselstyn, J. A. (2016). Local endemism and within-island diversification of shrews illustrate the importance of speciation in building Sundaland mammal diversity. Molecular ecology, 25(20), 5158-5173.

Dubey, S., Petra Nová, Vogel, P., Vladimír Vohralík. (2007). Cytogenetic and molecular relationships between zarudny's rock shrew (*Crocidura zarudnyi*; Mammalia: Soricomorpha) and Eurasian taxa. Journal of Mammalogy, 88(3), 706-711.

Dubey, S., Salamin, N., Ohdachi, S. D., Barrière P., Vogel, P. (2007). Molecular phylogenetics of shrews (Mammalia: Soricidae) reveal timing of transcontinental colonizations. Molecular Phylogenetics Evolution, 44(1), 126-137.

Dubey, S., Salamin, N., Ruedi, M., Barrière, P., Colyn, M., Vogel, P. (2008). Biogeographic origin and radiation of the Old World Crocidurine shrews (Mammalia: Soricidae) inferred from mitochondrial and nuclear genes. Molecular Phylogenetics and Evolution, 48(3), 953-963.

Dubey, S., Zaitsev, M., Cosson, J. F., Abdukadier, A., Vogel, P. (2006). Pliocene and Pleistocene diversification and multiple refugia in a Eurasian shrew (*Crocidura suaveolens* group). Molecular Phylogenetics and Evolution, 38(3), 635-647.

Eldridge, R. A., Achmadi, A. S., Giarla, T. C., Rowe, K. C., Esselstyn, J. A. (2018). Geographic isolation and elevational gradients promote diversification in an endemic shrew on Sulawesi. Molecular phylogenetics and evolution, 118, 306-317.

Esselstyn, J. A., Oliveros, C. H. (2010). Colonization of the Philippines from Taiwan: a multi-locus test of the biogeographic and phylogenetic relationships of isolated populations of shrews. Journal of Biogeography, 37(8), 1504-1514.

Esselstyn, J. A., Achmadi, A. S., Siler, C. D., Evans, B. J. (2013). Carving out turf in a biodiversity hotspot: multiple, previously unrecognized shrew species co-occur on Java Island, Indonesia. Molecular ecology, 22(19), 4972-4987.

Esselstyn, J. A., Timm, R. M., Brown, R. M. (2009). Do geological or climatic processes drive speciation in dynamic archipelagos? The tempo and mode of diversification in Southeast Asian shrews. Evolution: International Journal of Organic Evolution, 63(10), 2595-2610.

Esselstyn, J.A., Oliveros, C.H., 2010. Colonization of the Philippines from Taiwan: a multi-locus test of the biogeographic and phylogenetic relationships of isolated populations of shrews. Journal of Biogeography, 37, 1504–14.

Giarla, T. C., Esselstyn, J. A. (2015). The challenges of resolving a rapid, recent radiation: empirical and simulated phylogenomics of Philippine shrews. Systematic Biology, 64(5), 727-740.

He, K. , Li, Y. J. , Brandley, M. C. , Lin, L. K. , Jiang, X. L. (2010). A multi-locus phylogeny of Nectogalini shrews and influences of the Paleoclimate on speciation and evolution. Molecular Phylogenetics and Evolution, 56(2), 734-746.

Hutterer, R., Balete, D. S., Giarla, T. C., Heaney, L. R., Esselstyn, J. A. (2018). A new genus and species of shrew (Mammalia: Soricidae) from Palawan Island, Philippines. Journal of Mammalogy, 99(3), 518-536.

Kim, T. W., Kim, Y. K., Oh, D. J., Park, J. H., Kim, D., Adhikari, P., Oh, H. S. (2017). Complete mitochondrial genome of the Ussuri white-toothed shrew *Crocidura lasiura* (Insectivora, Soricidae). Mitochondrial DNA Part A, 28(2), 216-217.

Koh, H. S., Kartavtseva, I. V., Lee, B. K., Kweon, G. H., Yang, B. G., Heo, S. W., In, S. T. (2013). A preliminary study on genetic divergence of the Asian lesser white-toothed shrew *Crocidura shantungensis* (Mammalia: Soricomorpha) in mainland Korea, adjacent islands and continental East Asia: cytochrome b sequence analysis. Russian Journal of Theriology, 12(2), 71-77.

Lavrenchenko, Bannikova, L. A., Lebedev, A. A., V., S. (2009). Shrews (Crocidura spp.) endemic to Ethiopia: recent adaptive radiation of an ancient lineage. Doklady Biological Sciences, 424(1), 57-60.

Lin, X. D., Zhou, R. H., Fan, F. N., Ying, X. H., Sun, X. Y., Wang, W., ... Zhang, Y. Z. (2014). Biodiversity and evolution of Imjin virus and Thottapalayam virus in Crocidurinae shrews in Zhejiang Province, China. Virus research, 189, 114-120.

Liu, Z., Zhao, W., Liu, P., Li, S., Xu, C. (2016). The complete mitochondrial genome of Eurasian water shrew (*Neomys fodiens*). Mitochondrial DNA Part A, 27(4), 2381-2382.

Meegaskumbura, S., Meegaskumbura, M., Schneider, C.J. (2010). Systematic relationships and taxonomy of *Suncus montanus* and *S. murinus* from Sri Lanka. Molecular Phylogenetics Evolution, 55(2), 473-487.

Meegaskumbura, S., Schneider, C. (2009). A taxonomic evaluation of the shrew *Suncus montanus* (Soricidae: Crocidurinae) of Sri Lanka and India. Ceylon Journal of Science (Biological Sciences), 37(2).

Meegaskumbura, S., Meegaskumbura, M., Schneider, C. J. (2012). Re-evaluation of the taxonomy of the Sri Lankan pigmy shrew *Suncus fellowe sgordoni* (Soricidae: Crocidurinae) and its phylogenetic relationship with *S. etruscus*. Zootaxa, 3187, 57-68.

Meegaskumbura, S., Meegaskumbura, M., Pethiyagoda, R., Manamendra-Arachchi, K., Schneider, C. J. (2007). *Crocidura hikmiya*, a new shrew (Mammalia: Soricomorpha: Soricidae) from Sri Lanka. Zootaxa, 1665(1), 19-30.

Motokawa, M. (2004). Variation in the y chromosome of *Crocidura tadae kurodai* (Insectivora, Soricidae). Mammalian Biology, 69(4), 273-276.

Ohdachi, S. D. (2001). Mitochondrial cytchrome b sequence of *Crocidura kurodai*. Published Only in Database

Ohdachi, S. D., Hasegawa, M., Iwasa, M. A., Vogel, P., Oshida, T., Lin, L. K., Abe, H. (2006). Molecular phylogenetics of soricid shrews (Mammalia) based on mitochondrial cytochrome b gene sequences: with special reference to the Soricinae. Journal of Zoology, 270(1), 177-191.

Ohdachi, S. D., Iwasa, M. A., Nesterenko, V. A., Abe, H., Masuda, R., Haberl, W. (2004). Molecular phylogenetics of *Crocidura* shrews (Insectivora) in east and central Asia. Journal of Mammalogy, 85(3), 396-404.

Ohdachi, S. D., Kinoshita, G., Oda, S. I., Motokawa, M., Jogahara, T., Arai, S., Zu Min, M. (2016). Intraspecific phylogeny of the house shrews, *Suncus murinus*-*S. montanus* species complex, based on the mitochondrial cytochrome b gene. Mammal study, 41(4), 229-239.

Ohdachi, S., Masuda, R., Abe, H., Adachi, J., Dokuchaev, N. E., Haukisalmi, V., et al. (1997). Phylogeny of Eurasian Soricine shrews (Insectivora, Mammalia) inferred from the mitochondrial cytochrome b gene sequences. Zoologicalence, 14(Jun 1997), 527-532.

Omar, H., Hashim, R., Bhassu, S., Ruedi, M. (2013). Morphological and genetic relationships of the *Crocidura monticola* species complex (Soricidae: Crocidurinae) in Sundaland. Mammalian Biology-Zeitschrift für Säugetierkunde, 78(6), 446-454.

Sheftel, B. I., Bannikova, A. A., Fang, Y., Demidova, T. B., Alexandrov, D. Y., Lebedev, V. S., Sun, Y. H. (2018). Notes on the Fauna, Systematics, and Ecology of Small Mammals in Southern Gansu, China. Biology Bulletin, 45(8), 898-912.

Song, J. W., Kang, H. J., Gu, S. H., Moon, S. S., Bennett, S. N., Song, K. J., Klein, T. A. (2009). Characterization of Imjin virus, a newly isolated hantavirus from the Ussuri white-toothed shrew (*Crocidura lasiura*). Journal of virology, 83(12), 6184-6191.

Tu, F., Liu, S., Liu, Y., Sun, Z., Yin, Y., Yan, C., Zhang, X. (2014). Complete mitogenome of Chinese shrew mole *Uropsilus soricipes* (Milne-Edwards, 1871) (Mammalia: Talpidae) and genetic structure of the species in the Jiajin Mountains (China). Journal of natural history, 48(23-24), 1467-1483.
