# Supplementary material for: Multilocus phylogeny and cryptic diversity of white-toothed shrews (Mammalia, Eulipotyphla, Crocidura) in China
Source: BMC Evol Biol. 2020 Feb 14;20:29. doi: 10.1186/s12862-020-1588-8 (PMC7023792; doi:10.1186/s12862-020-1588-8)
Supplement: Supplementary file 4 — Additional file 4: Table S4. Primers used for PCR and sequencing. [file 12862_2020_1588_MOESM4_ESM.doc]

**Table S4 Primers used for PCR and sequencing**

| Primer name | Primer | Primers 5'--3' | Annealing Temp ° C | Citation |
| --- | --- | --- | --- | --- |
| cyt*b* | L14724_hk3 | GGACTTATGACATGAAAAATCATCGTTG | 47-50 | He et al., 2010 |
|  | H15915_hk3 | GATTCCCCATTTCTGGTTTACAAGAC |  |  |
| APOB | ApoBf | GCAATCATTTGACTTAAGTG | 47-50 | Dubey et al., 2007 |
|  | ApoBr | GAGCAACAATATCTGATTGG |  |  |
| BRCA1 | BRCA1f | TGAGAACAGCACTTTATTACTCAC | 47-50 | Dubey et al., 2006 |
|  | BRCA1r | ATTCATGTTCCATATTGCTTATACTG |  |  |
| RAG1 | Amp Rag1 F | AGCTGCAGYCARTACCAYAARATGTA | 48-52 | Murphy et al., 2001 |
|  | Amp Rag1 R1 | AACTCAGCTGCATTKCCAATRTCACA |  |  |

**References**

Murphy, W.J., Eizirik, E., O’Brien, S.J., Madsen, O., Scally, M., Douady, C.J., Teeling, E.,Ryder, O.A., Stanhope, M.J., de Jong, W.W., Springer, M.S., 2001. Resolution of the early placental mammal radiation using bayesian phylogenetics. Science 294, 2348–2351.

Dubey, S., Zaitsev, M., Cosson, J. F., et al. 2006. Pliocene and Pleistocene diversification and multiple refugia in a Eurasian shrew (*Crocidura suaveolens* group). Molecular Phylogenetics Evolution 38, 635–647.

Dubey, S., Salamin, N., Ohdachi, S. D., Barriere, P., Vogel. P., 2007. Molecular phylogenetics of shrews (Mammalia: Soricidae) reveal timing of transcontinental colonizations. Molecular Phylogenetics Evolution 44, 126–137.

He K, Li Y J, Brandley M C, et al. 2010. A multi-locus phylogeny of Nectogalini shrews and influences of the paleoclimate on speciation and evolution. Molecular Phylogenetics Evolution 56, 734–746.
